# Differential co-expression-based detection of conditional relationships in transcriptional data: comparative analysis and application to breast cancer

**DOI:** 10.1186/s13059-019-1851-8

**Published:** 2019-11-14

**Authors:** Dharmesh D. Bhuva, Joseph Cursons, Gordon K. Smyth, Melissa J. Davis

**Affiliations:** 1 Bioinformatics Division, Walter and Eliza Hall Institute of Medical Research, Parkville, VIC 3052 Australia; 2 School of Mathematics and Statistics, Faculty of Science, University of Melbourne, Melbourne, VIC 3010 Australia; 3 Department of Medical Biology, Faculty of Medicine, Dentistry and Health Sciences, University of Melbourne, Melbourne, VIC 3010 Australia; 4 Department of Clinical Pathology, Faculty of Medicine, Dentistry and Health Sciences, University of Melbourne, Melbourne, VIC 3010 Australia

**Keywords:** Gene regulation, Differential co-expression, Differential networks, Systems modelling, Immune infiltration, Breast cancer

## Abstract

**Background:**

Elucidation of regulatory networks, including identification of regulatory mechanisms specific to a given biological context, is a key aim in systems biology. This has motivated the move from co-expression to differential co-expression analysis and numerous methods have been developed subsequently to address this task; however, evaluation of methods and interpretation of the resulting networks has been hindered by the lack of known context-specific regulatory interactions.

**Results:**

In this study, we develop a simulator based on dynamical systems modelling capable of simulating differential co-expression patterns. With the simulator and an evaluation framework, we benchmark and characterise the performance of inference methods. Defining three different levels of “true” networks for each simulation, we show that accurate inference of causation is difficult for all methods, compared to inference of associations. We show that a *z*-score-based method has the best general performance. Further, analysis of simulation parameters reveals five network and simulation properties that explained the performance of methods. The evaluation framework and inference methods used in this study are available in the dcanr R/Bioconductor package.

**Conclusions:**

Our analysis of networks inferred from simulated data show that hub nodes are more likely to be differentially regulated targets than transcription factors. Based on this observation, we propose an interpretation of the inferred differential network that can reconstruct a putative causal network.

## Background

Comparative analysis of biological systems, where molecular changes are compared between conditions, has been instrumental in many advances of modern biological science. In particular, differential expression (DE) analyses have been used to elucidate meaningful variation between experimental conditions, disease states, and cell types. While DE methods have been essential to explore differences in the abundance of biomolecules (e.g. RNA), if two targets are simultaneously up- or downregulated, this does not provide any insight as to whether these changes are independent or coordinated. This has led to the development of gene-set analysis methods [1–3] where genes with a known association are simultaneously tested rather than individual genes; however, these methods rely on well-defined gene sets. Defining gene sets is not a trivial task due to the variable nature of biological systems (i.e. a gene set defined within one cell type may not generalise).

In contrast to exploring DE across conditions, there are also opportunities to extract functional information from the co-expression of genes [4] (i.e. concordant changes in transcript abundance) using gene regulatory network (GRN) inference methods such as WCGNA [5] and the *z*-score by Prill et al. [6]. As DE and co-expression-based GRN analyses complement each other in uncovering the functional relationships, there is growing interest in combining these two approaches. In contrast to these two well-established approaches, *differential co-expression* (DC) methods (also known as differential association [7], differential correlation [8–10], or differential network [11] methods) are increasingly being used to reveal dependencies between genes by identifying coordinated expression that differs across conditions, and this is likely to increase as larger data sets with hundreds or even thousands of samples increase in availability. These methods aim to identify changes in regulation of different gene expression programs across conditions, for example through common/shared signalling pathways and/or transcription factors (TFs), using changes in co-expression patterns. Such variation has been observed in cancer where programs such as proliferation are activated and/or apoptosis is repressed depending on the state and environment of the cancerous tissue [12]. This idea has been developed further by demonstrating that regulatory networks vary depending on the biological condition (i.e. the regulatory network can “re-wire”), contrary to the more traditional concept of static regulatory networks [13–15].

Both co-expression-based GRN analyses and DC analyses can be used to learn about regulatory networks; however, the inference made differs greatly. While co-expression analyses aim to infer regulatory relationships, DC analyses aim to identify conditional regulatory relationships. These two forms of analyses, and by extension inferences, are in fact orthogonal. The former focuses on concordant co-expression while the latter discordant/differential co-expression. Though these analyses help uncover regulatory mechanisms, the underlying inferences are not easily comparable. As such, in this benchmarking study, we focused on evaluating DC methods and interpreting results from their application.

This work begins with a review of established methods for differential co-expression analysis and discusses strengths and limitations identified in previous studies. To support unbiased benchmarking of methods, we have developed a R/Bioconductor [16] package, dcanr, which implements several published methods which previously lacked software implementations and uses existing libraries for methods where available. The dcanr package provides a unified interface to differential co-expression analysis methods and also includes an evaluation framework to benchmark differential co-expression inference methods in the context of regulatory network inference. To achieve this, we re-purposed a normalised Hill differential equation method for modelling signalling pathways from Kraeutler et al. [17] to model gene regulation and simulate expression data. This approach is attractive due to the relatively simple parameterisation process that preserves directional interactions from the regulatory network structure. This allowed us to propose a novel model for generating a true differential network, which we demonstrate is a more appropriate representation of what these methods are designed to infer from transcriptomic data.

Using a simulation framework based on this model, we used the F1 metric to rank method performance. Introducing variability in the simulations and measuring a diverse set of network and simulation properties allowed us to characterise variability in performance. By reverse engineering the generation of a true network for evaluation, our strategy also addresses the complex problem of interpreting differential networks. We then apply the most highly ranked differential co-expression analysis method to the study of TCGA breast cancer data and use the insights gained from simulations to propose a putative estrogen receptor (ER)-dependent regulatory sub-network. We highlight issues that are often encountered with differential co-expression analysis and identify the steps where caution should be exercised along with a brief discussion of the research required to improve their utility. Of note, we demonstrate that a number of “hub genes” identified within differential co-expression networks are likely to be strongly differentially regulated targets, challenging the classic interpretation of hubs as transcriptional “master regulators”.

## Results

### Survey of differential co-expression methods

Numerous methods have been developed for differential co-expression (DC) analysis, mostly over the past decade, and these can be categorised into four broad categories: gene-based, module-based, biclustering, and network-based methods.

Gene-based DC analysis methods identify genes that show changes in associations with other genes across the different conditions. They attempt to quantify the extent to which an individual gene is differentially associated with other genes rather than focusing on the nature, or mechanism, of that differential association. Such gene-level signal could arise from transcription factor (TF) loss of function at the protein level (including post-translational modifications), leading to a loss of regulation across some or all target genes [18]. Notably, if this TF had stable RNA abundances across conditions, it would not be identified from a DE analysis even though its targets may be differentially expressed. Gene-based methods would identify this TF as strongly differentially co-expressed, with its targets being weakly differentially co-expressed. Gene-based DC methods are further stratified into global and local gene-based DC methods. Global gene-based methods quantify differential association of a gene in the context of all other genes, that is, how differentially associated is the gene of interest to every other gene. Local gene-based methods differ in the context of genes whereby differential association is quantified against a subset of genes; for example, genes that are associated to the gene of interest in at least one condition. Global gene-based methods include DCglob [19], the N-statistic [20], differential PageRank centrality [21], and differential Eigen centrality [22]. Local gene-based methods include DCloc [19], DCp [23], DCe [23], DiffK [5], differential degree centrality [24], differential motif centrality [21], RIF [25], and metrics based on correlation vectors [26]. DiffRank is a hybrid of these classes where both local and global measures of differential association are computed for each gene [27]. Lichtblau and colleagues [22] evaluated a subset of methods and found that local and hybrid methods generally outperform global methods [22]. Correlation vector-based DC measures were also evaluated by Gonzalez-Valbuena and Trevino [26], demonstrating that methods which filter out weak correlations performed poorly compared to those that retain correlation information across all genes. In general, all gene-based methods compute pairwise correlations of gene expression or similar measures of association across the conditions independently and either use these directly or generate co-expression networks across the conditions for comparison. Independent computation of the association measures across the conditions assumes that errors are similar between conditions, and it should be noted that this would not be the case if the number of observations in each condition differ. Moreover, quantifying association differences does not account for estimation errors across both conditions.

Module-based analyses aim to incorporate information about gene connectivity. Under the assumption that genes within modules are cross-correlated, there will be a reduction in noise and increased statistical power. These methods can be classified by three criteria: (i) whether they detect differential co-expression within modules or between, (ii) whether modules need to be specified a priori or whether they are identified from the data, and (iii) the number of conditions across which comparisons can be made. Table 1 summarises these methods according to these properties. Often the choice of module comparison and module definition methods is interdependent. For example, between module comparisons with known modules assumes that all genes within the module are co-expressed, but many modules are context (i.e. disease state, treatment condition) specific and therefore co-expression may vary across conditions. In contrast, within module comparison with known modules limits the associations tested and thus associations with genes excluded from the module may be missed. The de novo identification of modules begins with construction of a differential co-expression network followed by module extraction. Due to the independence of modules extracted using de novo identification, both within and between module differential co-expression can be investigated. DICER and DiffCoEx have these properties and can thus be classified as network-based methods by discarding the module extraction phase. DCIM is the only method that discovers conditions without a need for prior specification and therefore may also be categorised as a biclustering method; biclustering methods simultaneously cluster across the rows and columns of a matrix—or both samples and genes in the context of transcriptomic data. The characterisation and performance of these methods has been reviewed in detail by Pontes and colleagues [42].

**Table 1:** Module-based differential co-expression methods

Method	Module comparison	Module definition	Number of conditions	Citation	Availability
DICER	Between	De novo	Multiple	[28]	jar file available from [29], dcanr v1.0.0 R/Bioconductor package
GSCA	Within	Known	Multiple	[30]	R package available from [31]
GSNCA	Within	Known	Two	[32]	GSAR v1.18.0 R/Bioconductor package
CoGA	Within	Known	Two	[33]	R package available from [34]
DiffCoEx	Both	De novo	Multiple	[35]	dcanr v1.0.0 R/Bioconductor package
CoXpress	Within	De novo	Two	[36]	R package available from [37]
dCoxS	Within	Known	Two	[38]	Supplementary material of the original publication
DCIM	Between	De novo	De novo	[39]	R package available from [40]
DiffCorr	Between	Known	Two	[41]	DiffCorr v0.4.1 R package available from CRAN

Finally, network-based methods aim to identify a differential co-expression network that contains associations that change across conditions. Most methods use correlation as a measure of association, although a subset use metrics or tests based on information theory, Gaussian graphical models, linear models, an expected conditional F-statistic, or generalised linear models. Table 2 lists these methods along with key properties. Network analysis identifies a single network of differences rather than independent co-expression networks across conditions. These networks contain information about specific differential associations between genes, and as such, they can be used to perform both gene-based and module-based analysis with appropriate summarisation methods (and we note that some methods such as DICER and DiffCoEx are listed in both categories).

**Table 2:** Network-based differential co-expression analysis methods

Method	Statistical method	Test	Number of conditions	Citation	Availability
*z*-score	Correlation	*z*-test	Two	[43]	dcanr v1.0.0 R/Bioconductor package
DGCA	Correlation	*z*-test	Two	[9]	DGCA v1.0.1 R package available from CRAN
Discordant	Correlation	*z*-test	Two	[10]	discordant v1.8.0 R/Bioconductor package
MAGIC	Correlation	Modulation test	Two	[11]	dcanr v1.0.0 R/Bioconductor package
DICER	Correlation	Permutation test	Multiple	[28]	jar file available from [29], dcanr v1.0.0 R/Bioconductor package
DiffCoEx	Correlation	Permutation test	Multiple	[35]	dcanr v1.0.0 R/Bioconductor package
EBcoexpress	Empirical Bayes + correlation	–	Two	[7]	EBcoexpress v1.28.0 R/Bioconductor package
Entropy (ENT)	Entropy based on correlation	Permutation test	Two	[44]	dcanr v1.0.0 R/Bioconductor package
FTGI	Generalised linear model	Chi-squared test	Multiple	[45]	dcanr v1.0.0 R/Bioconductor package
ECF	Expected conditional F	Permutation test	Multiple	[46]	COSINE v2.1 R package available from CRAN
COSINE	Expected conditional F	–	Multiple	[8]	COSINE v2.1 R package available from CRAN
GGM-based	GGM + posterior odds	–	Two	[47]	dcanr v1.0.0 R/Bioconductor package
LDGM	Latent differential graphical model	–	Two	[48]	dcanr v1.1.4 R/Bioconductor package
MINDy	Conditional mutual information	Permutation test	Two	[49]	MINDy module in GenePattern, dcanr v1.0.0 R/Bioconductor package

Network-based methods are attractive as modularity of the analysis framework facilitates multiple levels of DC analyses. Several methods use the *z*-test of correlation coefficients which computes a *z*-score after applying Fisher’s transformation to Pearson’s correlation coefficients. Differences across conditions can then be quantified as a difference in *z*-scores across conditions and modelled as a standard normal distribution. As the variances of transformed coefficients are pooled, the error estimate for the difference statistic is improved.

Some methods perform the same statistical tests to determine differential associations but apply alternative post-processing steps for categorisation and interpretation. For instance, DGCA and discordant both perform a *z*-test to identify the differential network, but additional analyses are applied to characterise interactions with respect to the conditions. Similarly, COSINE computes a network optimisation function using the ECF statistic which is optimised using genetic algorithms. In general, methods based on Pearson’s correlations, linear models, or graphical models are limited to identifying changes in linear relationships.

Network-based methods are flexible and can be used to identify both differentially co-expressed modules, as well as differentially co-expressed genes. In contrast, module- and gene-based methods cannot be used to reconstruct networks, due to the level at which association information is detected and summarised in the methods’ outputs. Since our goal here is to evaluate the ability of methods to reconstruct conditional regulatory networks, in the following evaluation, we focus on network-based methods only. Module and gene-based methods all have valuable applications [18, 24, 25, 35, 39], but are not suited for this specific task.

### Survey of evaluation methods

Given the numerous choices available, it can be challenging to select the most appropriate method for a given analysis. Early comparative evaluations proved useful in characterising the performance of gene regulatory network inference methods. In particular, the evaluation framework for the DREAM3 and DREAM4 challenges motivated the development of novel methods and helped to characterise methods based on motif discovery [6, 50]. Similar evaluations by Madhamshettiwar et al. [51] and Maetschke et al. [52] showed that topological properties of the true network and the type of experimental data used strongly influenced method performance. These comparisons used simulations to benchmark methods due to a lack of gold-standard test data; underlying regulatory networks have not been fully characterised in most complex organisms, and often these will change across different conditions and cell types [53]. Accordingly, while simulations may not capture the full complexity of biological systems, they provide a degree of control that is necessary for the comprehensive evaluations of DC methods.

An evaluation of DC methods by Kayano et al. [54] used simulations to compare methods: varying the data distribution, they were able to assess method performance in the presence of outliers and range biases across conditions. Another study by Siska and Kechris [55] assessed the ability of four alternative measures of correlation to identify differential associations and showed that Spearman’s correlation coefficient was a better and more stable indicator of associations for both count-level and transformed transcriptomic data from RNA-seq experiments. These previous studies only tested a small subset of available methods and evaluated their performance across a limited set of scenarios. Given this, we have developed a simulation framework that enables methods to be compared across a diverse set of scenarios.

To guide the development of DC methods and improve their adoption for bioinformatics analyses, it is also necessary to include a comprehensive evaluation framework to assess and compare different methods. An evaluation framework consists of three components: (i) a mathematical model of the system to simulate data, (ii) gold-standard/true data to evaluate predictions, and (iii) appropriate metrics to quantify the performance of different methods.

Two broad model classes can be used to simulate data: statistical models based on multivariate Gaussian mixture models [54], or dynamical systems models of gene regulation such as GeneNetWeaver [56] and SynTReN [57], which were previously used in the DREAM challenges. Multivariate Gaussian mixture models are simple and easier to use for generation of large data sets, but they are limited to simulating linear associations. Moreover, regulatory network structures cannot be incorporated into multivariate Gaussian mixture models therefore propagating effects of differential regulation cannot be modelled. Dynamical systems models have more flexibility to model non-linear associations; however, the increased number of model parameters can make them difficult to control. Differential co-expression data can be generated from these models by simulating knockouts or knockdowns on co-regulators in the network across a portion of the population. GeneNetWeaver and SynTReN can be used for this; however, current implementations pose a limitation in terms of flexibility. For example, users cannot easily specify knockouts or alternative initialisation parameters, making data simulation for co-expression problematic. Moreover, current implementations are in Java whereas most inference methods using these data are only available in R. Having an evaluation framework in the same environment as inference methods promotes comparisons against novel methods developed in the future.

Next, it is possible to generate a regulatory network structure and create alternative conditions such as gene knockout/knockdown and control for use with this simulation framework. Resulting data can be used for different inference methods, and the resulting network structures can be compared against the underlying truth network. The simplest true differential network would be the set of regulatory interactions directly influenced by the perturbation. An influence network that captures both direct and indirect associations may be a better true network, as changes in the network can propagate to downstream effects [58]. We note that Pe’er and Hacohen [13] also referred to such associations as regulatory influences rather than regulatory interactions further emphasising the idea of influence networks for these inference frameworks.

The final component is a metric to quantify performance. Numerous performance metrics exist, each possessing different properties, and previous evaluations have uncovered their relationships and assess their relevant usage scenarios [59]. The most commonly used metrics in co-expression and differential co-expression analysis are either based on the receiver operating characteristic curve (ROC), such as the area under the ROC curve (AUROC), or precision and recall [6, 50–52, 54, 60]. Under the assumption of sparsity in biological regulatory networks, metrics based on precision and recall are more appropriate than those based on the ROC curve [61].

### A flexible approach to simulating expression data from regulatory networks

Given the limited flexibility of existing network-based gene expression simulators, we developed a new framework for simulating expression data from realistic gene regulatory networks that allows genes to be either wildtype or knockdown across expression profiles in a simulation. This allows perturbation of input parameters and enables competing analysis methods to be compared across a diverse set of scenarios. Our simulator combines the method of Marbach et al. [59], which builds a biologically realistic set of regulatory interactions, with quantitative activation and repressor functions from Kraeutler et al. [17]. A network of direct regulatory interactions is first sampled from the *S. cerevisiae* (yeast) regulatory network using the method described in [62]. The total number of genes (nodes) can be pre-specified, as can the minimum number of regulators. For each regulatory interaction, an ordinary differential equation is generated that defines the activation or repression of the target gene’s expression as a function of the regulator gene’s expression. Where an individual gene is the target of multiple regulators, the activation and repression functions are assumed to combine multiplicatively corresponding to a logical *AND* gate.

The model can be used to generate expression levels for any number of genes and for any number of expression profiles. Expression levels are randomly generated for the input genes in each expression profile, allowing for the wildtype or knockdown status for each input gene in each profile and allowing for inter-gene correlation. Random noise is applied to the differential equations, and a non-linear equation solver is used to solve the steady-state levels of all other genes in the network given the expression of input genes. A small amount of additive noise is added to the final expression values. The simulator is available at [63]. Full mathematical details of the simulator are provided in “Methods”.

### Gene knockdowns induce differential associations between co-regulators and target genes

**Fig. 1:**
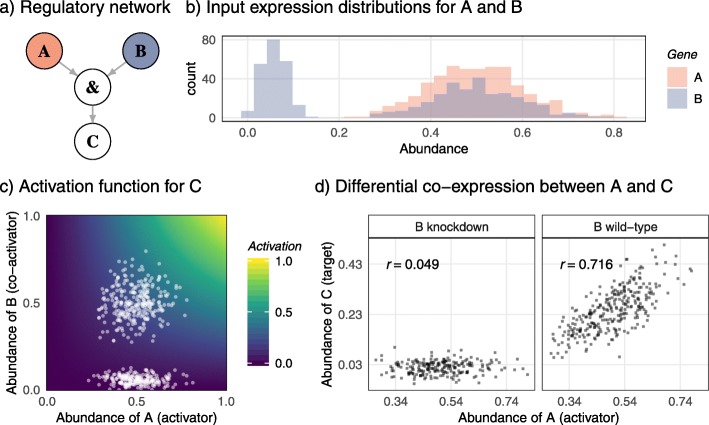
A simple regulatory network demonstrating differential co-expression. **a** Schematic of the regulatory network. Genes *A* and *B* are input genes and co-activate gene *C*. **b** Histograms showing the distribution of expression values for *A* and *B* across 500 simulated expression profiles. Gene *A* is always wildtype whereas gene *B* is knocked down in about half of the samples. **c** Scatterplot of expression values for *A* and *B*. Background shading shows the activation function generated by *A* and *B* used to model regulation of *C*. **d** Scatterplots of expression values for *A* and *C*, knockdown of *B* (left panel) and *B* wildtype samples (right panel). Gene *A* is highly correlated with *C* (*r* = 0.716) when *B* is at wildtype expression levels but uncorrelated with *C* (*r* = 0.049) when *B* is knocked down

Perturbing a gene by knocking down its expression in particular biological samples is a key experimental technique in functional genomics. Our simulations envisage a set 500 biological samples involving knockdowns for one or more of the input genes in the regulatory network. For each of the perturbed genes, some of the samples were generated to be wildtype with normal expression and the remainder were knockdown with abrogated expression for that gene. Knocking down a gene affects not only the expression of that gene but also the expression of its target genes and, indirectly, the expression of other genes via interactions across the regulatory network. Our focus in this article is on DC, which arises whenever the knockdown gene is a co-activator or a co-repressor of a target gene. In the common scenario that co-activators must cooperate to activate the target gene, but co-repressors can act individually, a gene knockdown tends to decrease the association between the co-activators and the target and tends to increase the association between co-repressors and the target. The simplest regulatory network to illustrate this phenomenon is that shown in Fig. 1a. We used our simulator to generate 500 expression profiles for genes *A*, *B*, and *C* assuming *A* and *B* to be co-activators of *C*. Gene *A* was always wildtype while gene *B* was knocked down in about half the samples, producing a unimodal distribution of expression values for *A* symmetric around 0.5 and a bimodal distribution of expression values for *B* (Fig. 1b). Figure 1c shows a bivariate plot of the expression values for *A* and *B* together with the activation function that *A* and *B* generate jointly to regulate the expression of *C*. The activation function for *C* takes on high values only when *A* and *B* both have high abundance (Fig. 1c). The correlation between *A* and *C* across all 500 samples is moderately positive (*r* = 0.246) but knockdown of *B* produces strong differential association. The correlation between *A* and *C* is very strong (*r* = 0.716) when restricted to *B* wildtype samples but essentially absent (*r* = 0.049) for *B* knockdown samples (Fig. 1d). An ordinary co-expression analysis therefore might miss the dependency between *A* and *C* whereas DC analysis would detect a strong signal.

### Determining differential co-expression for complex networks

**Fig. 2:**
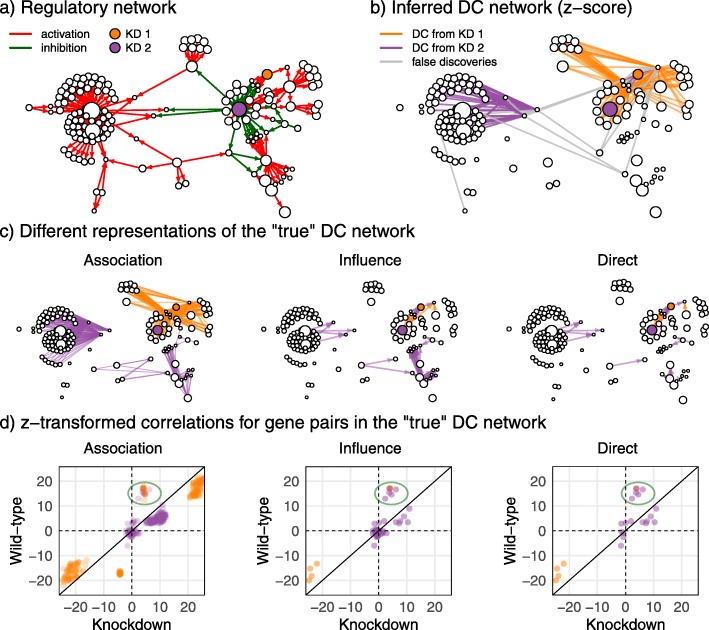
Differential co-expression analysis of an example network with 150 genes and 500 samples. **a** The regulatory network used to simulate the data and the two knockdown genes (KDs) (orange and purple nodes). **b** A differential co-expression (DC) network inferred from the simulated data using the *z*-score method. Interactions shown have significantly different correlations between knockdown and wildtype states (FDR < 0.1). Correct predictions for each knockdown as per the “true” *differential association* network are coloured respectively with false positives in grey. **c** Three representations of the true co-expression network obtained from a perturbation analysis of the regulatory network. Direct differential interactions are a subset of differential influences which are in turn a subset of differential associations. **d** Empirical *z*-transformed correlations for each interaction in the respective “true” networks. The association network shows a similar correlation profile to the direct and influence networks but with added points, as shown for example by the circled points

Larger and more complex regulatory networks show richer patterns of differential co-expression. Figure 2a shows the direct interactions for a randomly sampled network of 150 genes. The network includes 12 input genes, two of which were selected for perturbation and highlighted in purple and orange in the plot. Expression data was simulated from the network for 500 biological samples, with the two highlighted genes (KD1 and KD2) randomly assigned to normal or knockdown expression states in each sample (giving four possible combinations for each sample). Figure 2b shows the results of the *z*-score DC inference method applied to the expression data. For every gene pair and each knockdown gene, Pearson’s correlations and Fisher’s *z*-transform were used to test for a correlation difference between the wildtype and knockdown states of each gene knockdown. Correctly predicted differentially co-expressed edges resulting from each gene knockdown were coloured accordingly (purple or orange), and false positives were coloured grey.

Next, we analysed the regulatory network to determine which of the empirical correlation differences shown in Fig. 2b correspond to regulatory relationships that are genuinely perturbed by the knockdown gene. Such relationships are considered to represent “true” DC and the collection of such relationships is a representation of the “true” DC network. In Fig. 2c, we perform a perturbation analysis. We manipulate the network as a deterministic system without added noise. We perturb all input genes individually (including the two that are selected for perturbation in our simulation experiment—purple and orange) and determine which of the downstream genes are sensitive to the perturbation, that is, show a substantial alteration in response to perturbation of a given input gene. This analysis is necessary because not all genes downstream of an input gene are significantly responsive to perturbations of that input gene, especially in cases where the downstream gene has many other upstream input genes. Any input gene that shares a sensitive target with a knockdown gene should manifest DC with that target, as the strength of the association of the input and the target will be different in conditions where the knockdown gene is reduced in expression. We can extend the input gene results to downstream genes that are solely regulated by each input gene because they are tightly correlated with the input gene in the deterministic network. We call this set of DC interactions the *association DC network* (left panel of Fig. 2c). The association network includes non-causal gene-gene relationships that are “spurious” or “confounded” in the sense that the putative regulator is not upstream of the target gene in the regulatory network but is merely downstream of a causal regulator. Sub-setting the association network to gene pairs where the regulator is upstream of the target gene in the network produces the *influence DC network* (middle panel of Fig. 2c). Further sub-setting the influence network to gene pairs where the regulator is directly upstream (i.e. those in Fig. 2a) produces the *direct DC network* (right panel of Fig. 2c).

In essence, these three representations of the “true” network correspond to different levels of information propagation across the network. The direct network represents information flow from a node to an immediate downstream node. The influence network extends this to model information flow from a node to all sensitive downstream nodes for which there exists a path. The association network further includes information shared between nodes due to information flow from a common ancestral node. In this application, we are interested in the changes in information flow resulting from perturbations, and therefore differences in information flow across the regulatory network represent “true” DC networks and we compare all three levels in our evaluation.

Figure 2d shows the *z*-transformed correlation differences empirically observed from the simulated data for interactions in each of the true DC network. Some associations exhibit small changes in correlation across conditions and therefore may be difficult to detect with inference methods, but others show substantial differences in *z*-transformed correlations. Differentially co-expressed gene pairs in the influence and association networks cluster together with the direct association they are derived from (green circle) based on correlations between conditions. This shows that correlation profiles are retained despite the addition of edges to the differential influence network and the differential association network, thereby supporting these representations of true DC networks.

### Evaluation of inference methods using simulated data

We compared 11 differential co-expression inference methods by applying them to 812 simulated datasets (details in “Methods”). For the *z*-score method, we computed correlations using Pearson’s and Spearman’s methods therefore two sets of results were generated. These are hereafter referred to as *z*-score-P and *z*-score-S, respectively. Additionally, we evaluated DC networks generated from co-expression-based GRN methods by taking the difference between co-expression networks identified separately in each condition; WGCNA and a *z*-score method by Prill et al. [6] were used to generate these co-expression networks. Briefly, approximately 500 expression profiles were simulated from networks with 150 nodes and approximately 2–8 knockdowns performed. Some simulations could not be completed (*n* = 188), either due to an absence of co-regulation in the sampled source networks or a lack of observations in each condition. The resulting expression matrix (150 × 500) and *K* × 500 binary matrix for K knockdowns were used by the 11 inference methods to infer differential co-expression networks.

For each simulated regulatory network, true DC networks were determined from the mathematical model as demonstrated in Fig. 2. In particular, we propose the idea of an association network that includes causative associations captured by the influence network, as well as confounding associations resulting from similarity in abundance profiles. Algorithmic details are given in “Methods”. Performance of methods was evaluated using the F1 score, which was computed for all three representations of the true DC network (direct, influence, and association). Simulated data, inferred networks, and F1 scores for the 11 methods and 812 simulations in this report are available as a precomputed data set for import into the package (see “Availability of data and materials”). Functions in the dcanr (v1.0.0) R/Bioconductor package can be used to invoke inference methods, perform evaluations, and parse these data.

**Fig. 3:**
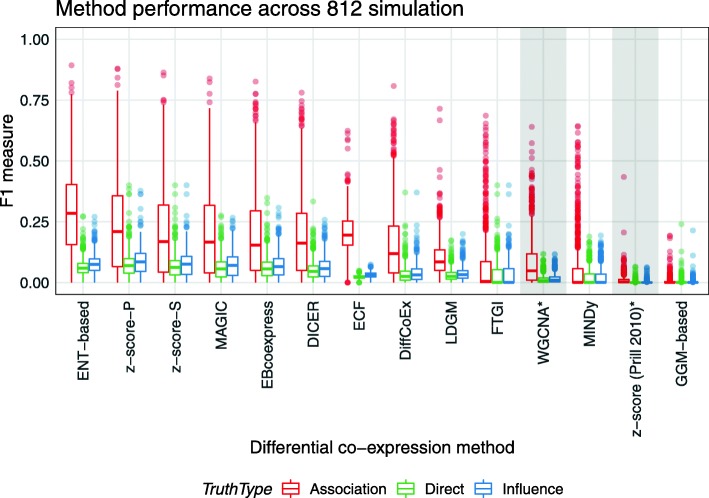
Most methods tend to infer the association DC network. Performance of 11 DC inference methods and 2 co-expression methods (highlighted in grey) across 812 different simulations with approximately 500 observations sampled. Performance is quantified using the F1 score and is computed for the three different representations of DC networks: direct, influence, and association. Methods are sorted based on the sum of their F1 scores across all simulations and truth networks. For co-expression methods, the difference of co-expression networks generated separately in each condition was taken as the DC network

Figure 3 summarises method performance across these differential networks. A striking observation is that methods tend to infer the differential association network better than the direct or influence DC networks. The example simulation shown in Fig. 2b also shows this property where the network inferred using the *z*-score is far closer to the association differential network. Overall, the performance of the entropy-based method (ENT-based) and the *z*-score calculated using Pearson’s coefficient (*z*-score-P) performed the best. The performance of *z*-score was slightly better than the entropy-based method for inferring the influence and direct networks; however, the latter performs better at inferring the association network. The GGM-based method, MINDy, and FTGI all performed poorly with the 90th percentile of F1 scores on the association network being lower than 0.25. The 90th percentile of F1 scores on the influence and direct networks were lower than 0.15 for all methods evaluated. As expected, most DC methods outperform co-expression methods (highlighted in Fig. 3) at DC inference. Though these methods work well in the task of co-expression analyses, simply taking the difference of co-expression networks does not successfully infer true DC relationships.

Varying the number of observations can influence method performance, therefore, to evaluate the effect of sample sizes, we ran 500 different simulations, sampling 500 observations first, and then sub-sampling 100 observations under the same settings. Methods were applied to the 396 simulations that converged and performance was quantified on how well they predict the differential association network. As expected, method performance generally dropped with lower sample numbers, except for ECF whose performance remained unaffected (Additional file 1: Figure S1). The F-measure is computed from precision and recall therefore we further investigated which of these quantities was mainly influenced by the reduction in sample numbers. The precision was reduced for all methods excluding ECF; however, the entropy-based method was somewhat robust to sample number and had a precision that was notably higher than all methods even with the reduced number of samples (Additional file 1: Figures S2 and S3). The recall of all methods except ECF dropped drastically. Though ECF was robust to the number of observations and generally had a high recall (median of 0.77), its overall performance was poor primarily due to a low precision (median of 0.11) reflecting large numbers of false positives. This analysis showed that the entropy-based method was better at dealing with low sample numbers compared to the other methods and that ECF may be an even better choice if the number of samples is very small. Reduction in recall is expected as the power of the analysis is affected when the number of samples are reduced which in turn would reduce the recall for most methods. The analysis also revealed the weakness of ECF being its precision, and recall being its strength. In general, correlation-based analyses require more samples than differential expression analysis and we recommend having at least 32 observations per condition to allow confident estimation of individual correlations greater than 0.3 (with a *p* value <0.05).

Though the entropy-based method performs relatively well across most simulations, inferences can be biased by sample size differences. Investigations into the effect of sample size differences revealed that the entropy-based method and MAGIC were influenced by differences in the number of observations across groups (see Additional file 1: Supplementary methods and Additional file 1: Figure S4). Biases in the number of samples in each condition are common in biological data, for example, the number of estrogen receptor-positive (ER^+^) samples in clinical breast cancer data is usually three times greater than the ER^−^ samples. In such cases, a method invariant to the differences in proportions is needed. Therefore, despite the slightly better performance of the entropy-based method, these results suggest that the *z*-score-based method is a better and more robust choice for generic applications, particularly when there is a class imbalance.

### Dissecting method performance

The complementary performance of some methods warranted an investigation into the properties that may be contributing to inference results. It was evident that some methods performed better than others in a subset of simulations, demonstrating that no method is in general the best, but rather most methods are better under specific scenarios. This highlights the need for users to consider the relative strengths and weaknesses of each method for their specific use case. To improve our understanding of the simulation parameters and properties that govern method performance, we generated summary statistics defining specific aspects of simulations. Performance was characterised for the *z*-score with Pearson’s coefficient. Simulations were classified based on the F1 score obtained from predicting the true DC network. Classification was performed using hierarchical clustering to group the simulations into five classes with varying degrees of “ability to be inferred”, such that, class label 1 represented those simulations where predictive performance of the *z*-score was best while class label 5 represented those where performance was poor. Summaries of the different properties were then investigated in these classes.

Analysis revealed that the average number of input regulators upstream of each differentially regulated target was the strongest determinant of performance (Additional file 1: Figure S5). The number of input genes also governed uncaptured variation in the data as evidenced by the negative association of performance with the number of inputs. As expected, multiple regulators increased the complexity of the signal observed for a target gene and this may have also reduced the association between input genes and their downstream targets, therefore obscuring any signal in the data used for inference. If instead multiple regulators were concordantly expressed, the amount of variation would reduce, thereby improving inference as shown when the variance of correlations of input genes was high (*μ* of correlations is 0; therefore, high *σ*^2^ means stronger correlations are observed between a subset of inputs). Biological systems are likely to exhibit such behaviour as regulation of genes required for specific processes results from a signalling cascade. Concordance of such targets and their transcription factors is therefore common. The number of perturbations applied per dataset was also negatively associated with inference performance which could be explained by convolution of the signal resulting from each independent perturbation. Weaker negative associations were observed with the density of the source regulatory network indicating that performance dropped as connectivity in the network increased. This may, in part, also be explained by increased convolution of the differential effects resulting from propagation of the signal. A less dense network would likely have a linear propagation effect where expression of a target relies on a small number of upstream regulators. The local clustering coefficient is indicative of the average number of cliques formed by nodes in the network. Since feedback loops are depleted from the original *S. cerevisiae* network, cliques would generally represent feedforward motifs. A larger local clustering coefficient would therefore represent the average number of feedforward loops per node. Interestingly, we observed that an increase in this metric resulted in better performance as indicated by the larger coefficients in the top two performing classes, perhaps reflecting the role of this motif in driving stable signalling.

Associations between classes and some of the summary statistics were of interest but so were the variables which did not influence inference performance. Our analysis revealed that inference performance by *z*-score method was invariant to the means of input genes, their variances, and the proportion of observations in each condition. This showed that performance was dependent on the structure of the regulatory network more than parameters of the simulation. However, it should be noted that these parameters could potentially impact performance when sampling the entire range, whereas our simulation procedure did not generate extreme observations and/or unusual distributions which may be seen in some real-world data.

### Hubs are targets rather than transcription factors

Several important observations were made using these simulations. First, the differential association network provides a better representation of the true network than the differential influence network and differential regulatory interactions (direct DC network). Without information on the directionality of associations, additional data, and accurate estimates of differential association magnitudes, it is practically impossible to infer the underlying regulatory network. Despite this, the differential network may help to infer some information about the structure of the underlying regulatory network. Structures in the regulatory network may present themselves in a different, yet consistent, form within the differential network, as demonstrated in Fig. 2. We tried to identify relationships between such structures by investigating differential association networks generated from specified regulatory networks across the 812 simulations. The strongest observation we made was that the node degree or connectivity of differentially regulated targets within the differential network was generally much greater than that of any other node. This challenges the classic interpretation proposed in many differential network analyses where high-degree nodes are proposed to be regulators/modulators [49]. The network in Fig. 2 shows this property for a single simulation where high-degree nodes within the differential network are indeed target genes in the regulatory network. To investigate this further, we generated the degree distribution of target genes and transcription factors across all 812 simulations. Only genes connected in the differential association network were analysed; target genes were defined as those with zero out-degree, and all other were genes considered as transcription factors (or general transcriptional regulators). These distributions are shown in the additional files (Additional file 1: Figure S6), with large differences in the mean log-transformed degree of target genes (2.55) and transcription factors (1.07). Conceptually, this could be expected as differentially regulated targets are associated with all upstream regulators and their co-expressed genes. Conversely, transcription factors would have a high degree only if they co-regulate many targets with other regulators (i.e. if they are master TFs).

### Applications to breast cancer

Differential co-expression analysis conditioned on the estrogen receptor (ER) status was performed on the TCGA breast cancer data using all DC methods, as described in the “Methods”. We filtered out any genes strongly associated with ER (with |correlations| > 0.5) to focus on those targets where ER is a co-regulator and not the sole regulator; this is analogous to filtering performed in the simulations. Five methods completed within the allocated computing resources (FTGI, DiffCoEx, *z*-score-P, *z*-score-S, and EBcoexpress). Scores for 5 more methods were computed but their statistical tests did not complete (DICER, entropy-based, GGM-based, ECF, MAGIC), and 2 methods (LDGM and MINDy) did not generate any results within the allocated time.

We first investigated the raw scores to assess similarity between all methods. Absolute scores from methods that use correlation-based measures were themselves highly correlated, with the exception of the entropy-based method (Additional file 1: Figure S7a). ECF and the GGM-based method produced the most distinct scores with very low to almost no association with scores from the other methods. Since statistical tests for some methods did not complete, we used the top 10,000 interactions with the highest absolute scores as a proxy for a predicted network. Overlap analysis of these networks reinforced the previous finding of concordance between inferences made using the correlation-based methods (Additional file 1: Figure S7b). The strongest overlap was between networks generated using the *z*-score with Spearman’s correlation coefficient and EBcoexpress. This observation was further validated by comparing the final predicted networks between these methods, which had both completed within the allocated execution time. We observed an adjusted Rand index (ARI) of greater than 0.7 for comparisons between DC networks generated from the correlation-based methods (EBcoexpress and *z*-score using either Pearson’s or Spearman’s correlation coefficients). FTGI and DiffCoEx generated distinct networks as evident from ARIs < 0.02 (Additional file 1: Figure S7c), likely due to differences in how each method calculates association (linear models and soft-thresholded correlation, respectively).

We then investigated structural properties of the networks from methods that fully completed. Degree distributions of all methods except DiffCoEx followed a power law indicating that these networks had a scale-free topology (Additional file 1: Figure S7d), while the DiffCoEx network had many nodes with high degree. While these results may be dataset-specific, we suspect it is because DiffCoEx is originally a module-based inference method. Networks generated using DiffCoEx are intended to be fed into the module detection phase and therefore tend to be densely connected and possess properties to facilitate module extraction. As the inference from both *z*-score methods and EBcoexpress were highly concordant, we decided to focus further investigations on a representative DC network: the DC network generated from the *z*-score with Spearman’s coefficient method.

**Fig. 4:**
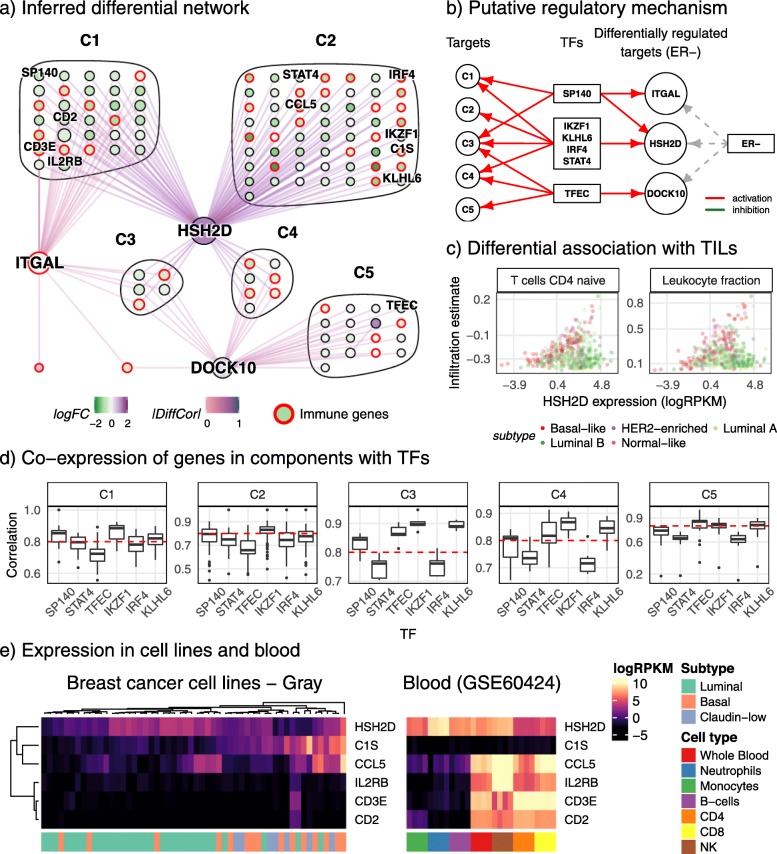
A DC sub-network in ER^−^ tumours is associated with lymphocyte infiltration. **a** The DC sub-network with candidate differentially regulated targets *DOCK10*, *HSH2D*, and *ITGAL*, and TFs *TFEC*, *SP140*, *IKZF1*, *KLHL6*, *IRF4*, and *STAT4*. Nodes are coloured based on log fold-change conditioned on ER status and edges coloured based on differences in correlations. Genes are clustered based on the target they are differentially co-expressed with. **b** A putative regulatory mechanism proposed from the DC network with insights gained from simulations. Dashed lines indicate a potentially indirect yet causal interaction. **c** Differential association of *HSH2D* with tumour-infiltrating lymphocytes (TILs) with infiltration estimated from a naïve T cell signature using singscore (left), and from H&E-stained slides (Saltz. Gupta, et al.). Associations indicate that *HSH2D* is a marker of lymphocyte infiltration specific to basal-like tumours. **d** correlations of genes in clusters C1-C5 with all transcription factors. The red line indicates a correlation of 0.8, showing stronger co-expression with TFs in the same cluster. **e** Expression of selected genes in cancer cell lines annotated with cancer sub-type and blood data annotated with immune cell type. Genes in the DC network have high expression in blood and are rarely expressed in cell lines

The *z*-score method resulted in a DC network with 178,487 differential associations between 8778 genes across ER^+^ and ER^−^ samples. As the resulting network was too large to investigate in full, we focused our analysis on the most statistically significant interactions (*p* value < 10^−10^). We selected a distinctive sub-network with strong negative *z*-scores, indicative of genes that show greater correlation across ER^−^ patients than ER^+^ patients. Three high-degree nodes were connected to the majority of the nodes within the sub-network, and thus a sub-network induced from these genes and their neighbours was analysed further. The resulting differential co-expression network is shown in Fig. 4a, centred on the high-degree nodes *HSH2D*, *DOCK10*, and *ITGAL*. Node colour is based on log fold-change of gene abundance between ER^+^ and ER^−^ tumours, and edge colouring reflects the difference in observed correlation coefficients, which could be considered as the effect size. Nodes were clustered based on their connectivity with the three putative targets.

Our observations from simulated data motivated the hypothesis that these three hubs are differentially regulated targets and that their associated TFs would be present within the connected nodes; all other nodes connected to the differentially regulated target would likely be targets directly regulated by the TF with no influence from ER. To test this hypothesis, we annotated TFs in the network using the union of genes in the gene ontology (GO) category GO:0003700 (“DNA-binding transcription factor activity”) and human TFs within the AnimalTFDB3.0 database [64, 65]. *IKZF1*, *IRF4*, *KLHL6*, *STAT4*, *SP140*, and *TFEC* were identified in the sub-network, but only *TFEC* was differentially associated with the hub gene *DOCK10*. These TFs have been labelled in Fig. 4a along with the three hubs. Next, we investigated whether these TFs were co-expressed with other neighbours of their differentially regulated target/hub, in this context perhaps representing direct regulation. Genes were clustered based on their connectivity with each target and labelled C1-C5, and then Spearman’s correlation of all nodes within a cluster was computed against each of the 6 TFs. Correlations for the resulting five clusters are presented in the boxplot in Fig. 4d. For the transcription factor *TFEC*, correlations were generally higher with other genes in the C5 cluster (with *DOCK10* as the only linked hub gene). According to our hypothesis, this would suggest that *TFEC* regulates or influences all genes in the C5 cluster, and differentially regulates *DOCK10*. Additionally, as the C3 and C4 clusters are connected to *DOCK10*, genes in these clusters are likely regulated by *TFEC* and other TFs. Similarly, differences in correlations were evident for C2, the *HSH2D*-only cluster where larger correlations were observed with *IKZF1* compared to other TFs. C5 genes also showed strong correlations with *IKZF1* and *KLHL6* which was explained by the high cross-correlation between these TFs and *TFEC* (0.81 and 0.87 respectively). Correlations between all genes were generally high which may be explained by the fact that the sub-network was enriched for genes involved in the regulation of immune response (GO:0050776; adj. *p* value < 2.11e−24) and T cell activation (GO:0042110; adj. *p* value < 3.03e−23). Indeed, the Ikaros family of transcription factors (e.g. *IKZF1*) have well-defined roles in lymphocyte differentiation and identity [66]. Genes differentially associated with two or more targets could possibly indicate common regulation by two TFs, for instance: genes in C4 exhibit stronger correlations with both *TFEC* and *IKFZ1* relative to other TFs; C3 genes were strongly associated with multiple TFs investigated (*TFEC*, *SP140*, *IKZF1*, and *KLHL6*); and C1 genes were associated with *SP140*, *KLHL6*, and *IKZF1*. Based on these observations, we have proposed a putative regulatory network in Fig. 4b. We note that upstream regulatory motifs were not investigated, and fully elucidating the mechanism would require further investigation and additional measurements across the system.

Two possible scenarios could explain the observed differential associations across conditions: differences in interactions between tumour cells and immune cells within the tumour microenvironment; or differences in the composition of the microenvironment. To investigate this, we analysed expression profiles for genes in the differential network within the Daeman et al. breast cancer cell line dataset which is devoid of immune infiltration, and a human blood cell dataset. All genes except for one were measured across both datasets. As expected, many genes were expressed solely in blood and not within tumour cell line models, although a few exhibited higher expression within both tumour cells and blood (Additional file 1: Figure S8). *C1S* was the only gene with high abundance in basal tumours that had almost no expression in luminal tumours or blood as shown in Fig. 4e. Interestingly, *HSH2D* showed expression in cell lines despite being previously reported as being solely expressed in haematopoietic cells [67]. A few genes including *IL2RB*, *CD3E*, and *CD2* were solely expressed in lymphoid lineage cells and not in myeloid lineages with a smaller proportion showing the opposite profile, further supporting the notion that many of these differential associations reflect changes in the relative immune composition within the tumour.

We further tested this hypothesis by scoring samples against immune signatures using the singscore method and a transcriptome-independent measure of immune infiltration (histopathology data). Both analyses revealed a significant difference in tumour infiltrating lymphocytes between the ER^−^ and ER^+^ tumours for all cell types using scores (*p* value <0.015 from a *t*-test), while for image-derived estimates most cell types showed significant differences (*p* value <0.035; excluding macrophages, *p* value =0.796; from a *t*-test, see Additional file 1: Figure S9). Correlations between the two estimation procedures were high (0.8–0.85) for lymphoid lineage signatures, therefore indicating the reliability of signature-based estimation. Interesting associations were identified with these scores and the genes in the differential network. Several genes, including *HSH2D*, *DOCK10*, and *ITGAL*, showed differential associations with immune scores, an example of which is shown for the naïve CD4^+^ T cell signature in Fig. 4c. These genes were positively associated with the score in basal-like tumours (which were mostly ER^−^), but no association was found for any of the other sub-types (Additional file 1: Figure S10). These genes were not present in any of the signatures tested; however, 40 genes from the differential network were and they did not exhibit a differential association (see *IKZF1* in Additional file 1: Figure S10). The result was also consistent with the leukocyte fraction estimated from imaging data (Fig. 4c), providing independent validation. Interestingly, only the associations of these genes varied with tumour infiltration and their abundances did not change significantly (i.e. they were not differentially expressed). These genes could be used as basal-like specific estimators of tumour infiltrating lymphocytes.

Our analyses support the hypothesis that the observed differential network can largely be attributed to differences in lymphocyte infiltration. We note, however, that the expression profile of *C1S* could possibly support the hypothesis that the observed DC network captures the relationship between tumour cells and infiltrating immune cells. *C1S* is a serine protease involved in the complement pathway and increased expression in basal-like cell lines may contribute to increased immune infiltration within ER^−^ cancers.

## Discussion

In this study, we evaluated the performance of 11 differential network inference methods, 8 of which were previously published but lacked usable implementations. We adapted a signalling network modelling method [17] to simulate gene regulatory network activity and generate synthetic expression data from known generative networks. The problem of precisely how gene expression is regulated contains many open questions, and it is questionable whether we yet have enough knowledge to come up with a truly accurate model. Here, we have instead generated a model that reproduces the observable output of the system (i.e. gene expression). Our simulations did not attempt to model binding events, stochasticity, translation, or post-translational modifications [56, 57], instead favouring simplicity to make the method tractable and flexible. Despite these strong assumptions, the model accurately captured the main biological property of interest to us in regulatory networks, namely signal propagation and its impact on gene expression. Moreover, most inference methods using transcriptomic data do not attempt to capture details of proteins or binding events, so our assumptions are not unwarranted in this context.

In order to simplify parameterisation of the simulations, we used the classification scheme proposed in SynTReN [57] where activation functions were split into five classes. The classification scheme restricted the range of activation functions such that extreme activation functions which are rarely feasible in biology are avoided. Studies have discouraged the use of random networks to represent biological networks due to large differences in topological structures [57, 62]; thus, we sampled networks from the *S. cerevisiae* regulatory network in a manner which retained biologically relevant motifs and network cliques [56]. Human regulatory networks were not used as they are sparsely identified. Since the human gene regulatory network (GRN) is sparsely identified yet, the known GRN can be thought of as being sampled from the full true human GRN. Sampling randomly (i.e. selecting random nodes) will result in differences in topological characteristics of the sampled network from the source network [57, 62]. Moreover, identification of the human GRN is mostly focused on genes/elements related to diseases which results in biased sampling of the true human GRN. The *S. cerevisiae* network is more comprehensively realised at this point; therefore, we assume that using it in simulations will result in more biologically accurate networks than using a larger but sparsely realised human network. The final modelling constraint was the set of logic functions used to model co-regulation. Here, we proposed logic functions derived from co-regulatory mechanisms that are representative of true biological regulation.

Additionally, we proposed a new parameterisation approach for input nodes which restricted variability at the population level. Traditional simulators such as GeneNetWeaver [56] and SynTReN [57] use uniformly distributed abundance values for input nodes thereby assuming all samples are independent. Real biological data tend to contain sample populations which exhibit similar behaviour with minor variations (e.g. within tumour sub-types); this is better modelled with a normal distribution. Moreover, we consider input genes to be weakly dependent, as we note that in biological systems the assumption of complete independence across all genes is invalid. Our parameterisation accounted for both the above observations in order to exclude extreme and possibly rare instances.

Observations from our simulations have led us to propose a novel interpretation of the true differential co-expression network, along with the previously proposed influence network [13, 14] and the classically used direct network. Of the three representations of the true network, we show that the differential association network is a better representation of what DC methods infer. Intuitively, this made sense as methods set out to infer *differential co-expression* networks rather than *differential regulatory* networks. While it is common to assume that networks inferred by DC methods can be interpreted as a regulatory network, our analysis demonstrates that this is not the case and that network topologies should be interpreted with this distinction in mind. We propose that this distinction should also apply to the evaluation of general co-expression network inference methods. Previous evaluations of network inference with co-expression methods have used the regulatory network as the true network for evaluating performance [50–52].

Given the results presented here, we propose that methods detecting co-expression should use the association network as the true network for benchmarking. We have shown this to be the case for all differential co-expression network methods examined here, despite differences in performance. It is also evident that if we use a direct or influence network as the true network, the usefulness of all methods is largely underestimated. In other words, while no method reconstructed the generative regulatory network well (in agreement with our previous work [51, 52]), most methods could reconstruct a co-expression network with reasonable accuracy. We found that the simple *z*-score method performed the best in benchmarking. Performance of co-expression methods such as WGCNA and the *z*-score method by Prill et al. [6] was poor in the task of DC inference as could be expected given their development for an alternative application. This demonstrates the need for specialised methods for DC analysis and should discourage the construction of DC networks using the difference between separately inferred co-expression networks, even when the best co-expression analysis methods are used. Additionally, this observation suggests that DC analysis methods, at gene, module, or network-level resolution, should refrain from performing inference across the conditions independently and should instead jointly estimate differences between conditions. Validating the choice of the true network through simulation allowed us to identify structures in the differential network that were indicative of the underlying regulatory network structure. One striking outcome of this detailed analysis is that hub genes in DC networks are more likely to be targets than regulators, contrary to the common assumption that a hub gene is likely to be a regulator. This can be used to guide downstream analyses, enhance the interpretation of co-expression networks, and support the identification of important nodes in the generative regulatory network.

Knowing that inference methods identify an association network, the challenge becomes interpreting these results with respect to the underlying biology. The aim of many transcriptomic analyses is to learn about the underlying biological system, and in the context of differential co-expression analysis, this is the regulatory network driving observed patterns within the data. Completely elucidating the regulatory network with only multifactorial transcriptomic data is difficult, as influences and direct regulatory relationships are a subset of the inferred network with non-unique mappings. The key required step here would be inference of directionality of edges. Given a directed *differential* co-expression network and a directed co-expression network (identifying shared behaviour), an influence network [13, 14] could be derived, providing some insight into the true regulatory network. Directionality can either be inferred from time-series and/or systematic perturbation experiments, or from knowledge bases such as TF-target binding databases, although the latter may limit novel discoveries. Though the aim of complete network inference may not be feasible with transcriptomic data alone, higher-order tasks are still possible, such as identifying dysregulated processes. For example, module identification following differential co-expression network inference may identify perturbation in cellular processes.

In this benchmarking study, we have focused on the analysis of differential co-expression between two conditions. The scenario where DC is detected across multiple conditions is an interesting one; however, it presents many complexities. Of the methods examined here, only five (ECF, COSINE, DiffCoEx, FTGI, and DICER) allow for multiple conditions. With the exception of ECF and COSINE, they do so by constructing a pairwise comparison, where each group is compared against the average of the others, or a selected reference condition. ECF and COSINE perform a series of pairwise comparisons and aggregate the statistic, in a process analogous to ANOVA. Thus, there is a clear need for the development of new methods that deal with truly multiple comparisons in a way that preserves information about the nature of the differences across conditions.

Our differential co-expression analysis of breast cancer data using estrogen receptor (ER) status as the differential condition revealed a sub-network related to immune activity. Combining the differential network with a basic co-expression analysis and differential expression analysis, we characterised the differential network and proposed a putative regulatory mechanism involving transcription factor regulation specific to ER^−^ tumours. We further showed that differentially regulated targets were also differentially associated with tumour infiltrating lymphocytes, suggesting a potential use in estimating lymphocytic infiltration for basal-like tumours. Based on these findings, we conclude that changes were likely observed due to differences in the tumour microenvironment across conditions. ER status is a complex factor, with numerous molecular differences in addition to changes in regulatory mechanisms. For instance, differences in lymphocyte infiltration have also been previously observed [68]. Consequently, the condition used to generate the differential network is likely to be confounded with co-occurring phenotypic changes, limiting interpretation from bulk RNA-seq data alone. Single-cell RNA-seq data might be useful in such a scenario, or inference methods based on partial correlations could account for such effects; however, neither type of method has yet been developed. Generally, we recommend acknowledging the fact that conditions are rarely independent in real data and accounting for this when interpreting results from a differential analysis.

Finally, we showed the application of signatures/gene sets in differential association analysis with the differential associations observed between *HSH2D* and the naïve T cell signature. Signatures have been used in a similar context to identify conditions that are otherwise difficult to measure [69]; however, their application in differential co-expression analysis remains unexplored. Furthermore, we showed that differential associations with non-transcriptomic data also exist and they too can be identified. Both cases present interesting avenues for future applications of these methods.

## Conclusion

Differential co-expression (DC) analysis is a powerful tool for understanding differences between samples belonging to different groups. Here, we have undertaken a benchmarking study to explore the performance of 11 DC analysis methods, and we provide implementations for eight of these in the dcanr R/Bioconductor package associated with this work. Across our simulations, we found the *z*-score method to have the best performance. Our simulation framework allowed us to focus not only on evaluating DC network inference methods, but also on the problem of how resulting networks can be interpreted in the context of their generative regulatory networks. We show that common interpretations of inferred network topology are often flawed and that a deeper understanding of the relationship between co-expression networks and regulatory networks is not only possible, but also critical to the accurate interpretation of the results of such methods.

## Methods

### Random sampling of network topologies

Networks in this study were sampled from *S. cerevisiae* (yeast) regulatory networks obtained from the SynTReN v1.2 supplementary data in simple interaction format (SIF) [70]. The SynTReN file provides a directional regulatory network containing 690 nodes/genes and 1094 edges with annotations for edge types. The edge type represents the type of regulatory relationship: activation, repression or both (dual). In our simulations, any dual interaction was reset to a repressor. Networks with 150 nodes (genes) were sampled using the method described in [62], which ensures most network properties of the original network are retained in the sampled network. A sampling bias was introduced to ensure at least 10 input genes (genes without regulators) were selected and stochasticity was set at *k* = 25%. All randomly sampled networks have a single component, in that a path exists from each gene to every other gene (discounting directionality of edges).

### Mathematical model of gene regulation

The normalised-Hill differential equations from Kraeutler et al. [17] were re-purposed to model activation/repression of a gene by a set of regulator genes. The following equation was used to model the activation of a gene *B* by a single regulator gene *A*:

$$ \frac{dB}{dt}={f}_{\mathrm{act}}\left(A,{\mathrm{EC}}_{50}^{AB},{n}^{AB}\right)-B $$

Here *f*_act_ is the activation function, *A* is the relative abundance of gene *A*, *B* is the relative abundance of gene *B*, $\mathrm{E}{\mathrm{C}}_{50}^{AB}$ is the abundance of gene *A* required for half-maximal activation of gene *B* and *n*^*AB*^ is the Hill constant used to specify linearity of the activation function. The activation function is defined by

$$ {f}_{\mathrm{act}}\left(A,\mathrm{E}{\mathrm{C}}_{50}^{AB},{n}^{AB}\right)=\frac{\beta {A}^{{\mathrm{n}}^{AB}}}{K^{n^{AB}}+{A}^{n^{AB}}} $$

with

$$ \beta =\frac{\mathrm{E}{{\mathrm{C}}_{50}^{AB}}^{n^{AB}}-1}{2\mathrm{E}{{\mathrm{C}}_{50}^{AB}}^{n^{AB}}-1} $$

and

$$ K={\left(\beta -1\right)}^{\frac{1}{n^{AB}}} $$

All abundance values are in the range [0,1].

Repression can be modelled using the activation function $1-{f}_{\mathrm{act}}\left(A,\mathrm{E}{\mathrm{C}}_{50}^{AB},{n}^{AB}\right)$. Co-activation of a gene by two regulators, *A*_1_ and *A*_2_ is modelled using the activation function ${f}_{\mathrm{act}}\left({A}_1,{\mathrm{EC}}_{50}^{A_1B},{n}^{A_1B}\right)\times {f}_{\mathrm{act}}\left({A}_2,{\mathrm{EC}}_{50}^{A_2B},{n}^{A_2B}\right)$. The activation function for multiple regulators is both commutative and associative which is useful considering the fact that the yeast network has a node with 13 regulators. The EC_50_ and *n* parameters define the relationship between each regulator and its target. To restrict to *linear-like* activation functions [57], we sampled EC_50_ from the range [0.4,0.6] and *n* from the range [1.01,1.70]. A rate equation is generated for each target gene. Steady-state levels for all genes were obtained using a non-linear equation solver.

### Simulating expression data from a regulatory network

Expression values were simulated from each regulatory network. Expression values for the input genes were generated from a truncated multivariate normal distribution on the interval [0,1] using a random mean vector and covariance matrix. The normal distribution means were sampled from a *B*(10,10) beta distribution for wildtype genes or *B*(10,100) for knocked down genes. The normal distribution variances were sampled from *B*(15,15) and then scaled by min(*μ*,(1 − μ))/3 where *μ* is the mean; scaling ensured that support for the normal distributions was concentrated within the range [0,1]. The correlation matrix for non-knockdown input genes was generated using the C-vine algorithm with partial correlations sampled from a *B*(5,5) distribution on [−1,1] [71]. Knockdown input genes were generated to be independent of other genes to avoid confounding the differential signal. The mean vector and covariance matrix of the multivariate normal distribution was held constant across all realisations of each network, apart from the switches from wildtype to knockdown states for selected input genes.

Once the abundances of all standard and knockdown input genes are generated, the expression values of all other genes are determined by solving for the steady state of the system of differential equations. Two types of noise are added to the simulated data to model experimental and biological noise. The activation functions were multiplied by lognormal random variables with *μ* = 0 and *σ* = 0.05 before solving the differential questions. After solving the differential equations, Gaussian noise with *μ* = 0 and *σ* = 0.05 was added to the expression values.

### Deriving the “true” differential association network from a model

This approach uses perturbations to determine the true differential association network for each simulation and allow performance evaluation. As noted above, a subset of genes is defined as input nodes and their abundances are sampled rather than calculated through network simulation. The expected value of each input node was independently perturbed with a 25% reduction resulting in an abundance of *μ*_*i*_ ∗ (1 − 0.25), and where these input nodes correspond to knockdown targets, the “wildtype” mean is used. Resulting changes in the abundance of other genes are then calculated and “perturbation sensitivity” values are calculated, defined here as the relative expression change in the target gene divided by 0.25 [17]. Absolute abundance values less than 0.001 are set to 0 to account for numerical inaccuracies encountered while solving for steady states. It should be noted that if *linear-like* activation functions are used the sensitivity calculation is invariant to the size of the perturbation. Gene pairs with dependencies are then identified by applying a threshold of 0.01 to absolute perturbation sensitivity values; this results in a binary *sensitivity* matrix where each entry indicates whether a gene is affected/sensitive to perturbation to another gene. At this stage, the network of associations represented by the sensitivity matrix is considered as the influence network [14]. This matrix is then used to infer the three representations of the “true” DC network using the algorithm described in the Additional file 1: Supplementary Methods.

### Simulation setup for evaluations

Method performance was evaluated across 1000 simulations. Simulated networks with 150 nodes were sampled from the *S. cerevisiae* network and approximately 500 expression profiles simulated from the network, resulting in 150 × 500 expression matrix. Some simulations resulted in fewer expression profiles due to the steady-state not being solved. The genes to knockdown per simulation were sampled from the input nodes with a probability of *ρ* (i.e. from a binomial distribution). Then, for each knockdown gene, expression profiles with the knockdown followed a binomial distribution Binomial(*p* = *ρ*, *N* = 500) where the proportion of such profiles were sampled from the uniform distribution Uniform(0.2,0.8). With *K* gene knockdowns being performed in a simulation, a *K* × 500 binary matrix was used to represent conditions. The expression matrix and condition matrix were then used by inference methods to predict a differential co-expression network conditioned on each knockdown.

### Summary statistics of simulations

Network properties and simulation parameters define each simulation. Summary statistics for 16 important characteristics were calculated for each simulation, 5 representing parameters of the dynamical systems model and 11 representing the network structure. Some properties map one-to-one with each simulation while others have a one-to-many relation (Table 3). Network properties were calculated using the igraph (v1.2.1) R package (available from CRAN) [72].

**Table 3:** Network and model properties calculated to characterise simulations

Label	Description	Type	Mapping
Avg num input TFs	Average number of input genes co-regulating the differentially regulated target.	Network	1-to-1
Clust coef (g)	Ratio of cliques over all possible cliques in the network. Large values are indicative of small-world networks. Calculated on the undirected regulatory network.	Network	1-to-1
Clust coef (l)	Average of clustering coefficients calculated per node. Calculated on the undirected network.	Network	1-to-1
Density diffnet	Network density of the true differential network. Here the differential association network is used.	Network	1-to-1
Density source	Network density of the source regulatory network. Density is calculated as the ratio of observed edges over the total number of possible edges (*N* choose 2) for *N* edges.	Network	1-to-1
Diameter	Calculated as the longest of all shortest paths in the network and is indicative of the linear size of the network. Calculated on the undirected network.	Network	1-to-1
Eigen centrality	Eigen centrality of each perturbed (knockdown) node. Calculated on the undirected network.	Network	1-to-many
Input means	Mean value of the distribution each input gene	Model	1-to-many
Input vars	Variance of the distribution of each input gene	Model	1-to-many
KD sample props	The smaller proportion of the population resulting from a knockdown. 0.2 if the proportions are 0.2 and 0.8.	Model	1-to-many
Num co-targeted	Number of differentially regulated targets	Network	1-to-1
Num inputs	Number of input nodes in the source regulatory network.	Network	1-to-1
Num KD genes	Number of perturbed genes (knockdown).	Model	1-to-1
Num TFs	Number of regulators in the source regulatory network (both inputs and downstream).	Network	1-to-1
Radius	Minimum eccentricity of any node where eccentricity of a node is the shortest distance to the farthest node. Calculated on the undirected network.	Network	1-to-1
Var of input cors	Variance of the correlations between relative abundances of input genes.	Model	1-to-1

### Inferring differential co-expression networks

Some of the methods examined here had available R package implementations (Table 2), although most were either unavailable or available on other platforms. For the graphical Gaussian model (GGM)-based method, models were fit using the GeneNet (v1.2.13) R package (available from CRAN) with the remaining analysis performed as described by Chu et al. [47] and implemented in our R/Bioconductor package dcanr (v1.0.0). The minimum and maximum values for the regularisation parameter for LDGM were computed as described by Tian et al. [48]. The parameter was tuned within this interval such that the number of edges in the resulting network matched the average number of edges in the “true” differential association networks resulting from each knockdown. For a knockdown resulting in 100 differential associations, the regularisation parameter would be selected such that the DC network had close to 100 edges. Binary search was performed in the interval to optimise for this parameter for up to 50 iterations. If the parameter was not optimised, the value that minimised the difference between the observed and expected number of edges among the 50 iterations was chosen.

Remaining methods were implemented to score and test independent associations, although additional downstream analyses such as module detection or filtering of significant associations based on heuristics were not implemented. We aimed to benchmark how well each method quantified independent differential associations. As such, downstream analyses such as module extraction by DICER and DiffCoEx and “minimum modulator support” by MINDy were not performed. Additionally, the output of all methods, excluding EBcoexpress, is a set of *p* values for all possible gene pairs. Interface functions to all existing implementations were developed to allow further comparison of results. MINDy inferred directional networks; therefore, for each edge, the maximum statistic in either direction was chosen to be representative of its score. This made sure all inferred networks were undirected. Where permutation tests are required, five permutations of the data were computed, and the statistic was pooled for each perturbation. These *p* values were then corrected for multiple testing using the Benjamini-Hochberg procedure [73] for each perturbation/condition. EBcoexpress produces posterior probabilities; therefore, these were used directly. An FDR cut-off of 0.1 was applied for each method excluding EBcoexpress, for which a maximum a posteriori probability cut-off of 0.9 was applied producing the final binary predictions of edge absence/presence. Prior to inference, genes only regulated by the knocked down gene were filtered out to maintain conditional independence. These were selected from the perturbation analysis as genes that were sensitive to the knocked down gene only and no other input gene. Precision, recall, and the F1 score were then computed for each method.

Differential co-expression inference was also performed using co-expression-based GRN analysis methods. Co-expression networks were generated in the knockdown and wild-type conditions independently, and the difference network between the two conditions (i.e. non-overlapping edges) formed the DC network. The WGCNA (v1.68) R package (available from CRAN) was used to run the WGCNA algorithm with default parameters. The co-expression network was generated by selecting all edges with a weight greater than 0.05. The *z*-score method by Prill et al. [6] was implemented as originally described. A two-tailed *z*-test was applied for this method, and *p* values were adjusted using the Benjamini-Hochberg procedure [73]. An FDR threshold of 0.1 was applied to result in the final co-expression networks.

### Implementation of the evaluation framework

Simulations and analysis were performed using R. Simulations and regulatory networks are encoded in S4 classes to ensure code stability and information organisation. The MASS (v7.3-50) R package (available from CRAN) [74] is used to sample data from multivariate normal distributions. The non-linear equation solver in the nleqslv (v3.3.1) R package (available from CRAN) is used to solve the differential equation system. Parallelisation is achieved using the foreach (v1.4.6) and doSNOW (v1.0.16) R packages (available from CRAN). Inference methods used in this study along with the evaluation framework are available in the dcanr (v1.0.0) R/Bioconductor package. Data from the 812 simulations performed along with inferred networks and F1 scores for the 11 methods are available as a separate file (see “Availability of data and materials”). Source code for performing the simulations is available at [63].

### TCGA breast invasive carcinoma analysis

TCGA breast invasive carcinoma (BRCA) HTSeq count-level RNA-seq data were downloaded from the genomic data commons (GDC) using the TCGAbiolinks (v2.8.2) R/Bioconductor package [75] with male and FFPE samples discarded. Genes with low expression (CPM < 2 across more than 50% of samples) were filtered out along with non-protein coding genes. TMM normalisation was performed on filtered data and logFPKMs computed using the edgeR (v3.22.3) R/Bioconductor package [76]. Gene lengths for computing logFPKMs were calculated as the summed length of all exons from Gencode v22 annotation files. We adapted code from the SingscoreAMLMutations (v1.0.0) R/Bioconductor package to download and process TCGA data [77]. Samples without annotation for ER status or samples with a “Indeterminate” ER status were discarded. Genes with an absolute correlation greater than 0.5 with the ER gene (*ESR1*) were removed and differential co-expression analysis was performed on the remaining data, conditioned on the ER status.

All methods were applied to the dataset with the same parameters as those used for simulated data. An adjusted *p* value threshold of 1 × 10^− 10^ was applied to generate the DC network. A threshold of 1 × 10^− 10^ was applied on the posterior probabilities generated by EBcoexpress. The regularisation parameter for LDGM was tuned to produce a network with 4700 edges; the average of the number of edges resulting from the two *z*-score executions (with Pearson’s and Spearman’s coefficient). As some methods were computationally intensive, we allocated 20 processors per method and allowed for a maximum wall time of up to 7 days (up to 3360 CPU hours per method dependent upon the efficiency of parallelisation). Network visualisation was performed using Cytoscape (v3.6), and network analysis used both Cytoscape and the igraph R package (available from CRAN). The RCy3 (v2.0.86) R/Bioconductor package provides a simple, complete interface between R and Cytoscape and was used to load and analyse networks across the two platforms.

The Daeman et al. breast cancer cell line RNA-seq data (GSE48213) [78] and sorted blood cell data (GSE60424) [79] were processed as described in [80]. Additionally, a processed microarray dataset of sorted blood dataset was used (GSE24759) [81]. Immune signatures [82] were used to estimate tumour infiltration from transcriptomic data using the singscore method [80] implemented in singscore (v1.4.0) R/Bioconductor package. Estimates from the analysis of H&E-stained slides [83] were used as an independent measure of tumour-infiltrating lymphocytes.

## Supplementary information

**Additional file 1. **Supplementary methods. **Figure S1.** F1 measure of inference methods across 396 simulations with either 500 or 100 observations. **Figure S2.** Precision of inference methods across 396 simulations with either 500 or 100 observations. **Figure S3.** Recall of inference methods across 396 simulations with either 500 or 100 observations. **Figure S4.** Score profiles for the different methods without (top) and with (bottom) imbalanced samples in each condition. **Figure S5.** Classes were determined by hierarchical clustering of the F1 score of the *z*-score with Pearson’s coefficient method across 812 simulations with 1 representing simulations where methods the *z*-score performed well the best and 5 where performance was poor. **Figure S6.** Degree distribution of target genes and transcription factors. **Figure S7.** Comparing differential co-expression scores and networks generated using different inference methods. **Figure S8.** Expression of genes from the ER dependent differential co-expression network in breast cancer cell lines and sorted blood datasets. **Figure S9.** Immune infiltration in the TCGA breast cancer cohort estimated from the RNAseq data using the signatures from CIBERSORT using the singscore gene set scoring method (top) and from image analysis of H&E stained slides of samples by Saltz et al. for the 7 cell types (bottom). **Figure S10.** Association between naïve CD4+ T cell infiltration estimates and selected genes from the differential co-expression sub-network containing immune associated genes.

**Additional file 2.** Review history.

## Data Availability

Data supporting the conclusions of this article is available in the University of Melbourne figshare repository https://melbourne.figshare.com/articles/812_simulated_expression_datasets_for_differential_co-expression_analysis/8010176 [84].
