# Supplementary material for: Differential co-expression-based detection of conditional relationships in transcriptional data: comparative analysis and application to breast cancer
Source: Genome Biol. 2019 Nov 14;20:236. doi: 10.1186/s13059-019-1851-8 (PMC6857226; doi:10.1186/s13059-019-1851-8)
Supplement: Supplementary file 1 — Additional file 1. Supplementary methods. Figure S1. F1 measure of inference methods across 396 simulations with either 500 or 100 observations. Figure S2. Precision of inference methods across 396 simulations with either 500 or 100 observations. Figure S3. Recall of inference methods across 396 simulations with either 500 or 100 observations. Figure S4. Score profiles for the different methods without (top) and with (bottom) imbalanced samples in each condition. Figure S5. Classes were determined by hierarchical clustering of the F1 score of the z-score with Pearson’s coefficient method across 812 simulations with 1 representing simulations where methods the z-score performed well the best and 5 where performance was poor. Figure S6. Degree distribution of target genes and transcription factors. Figure S7. Comparing differential co-expression scores and networks generated using different inference methods. Figure S8. Expression of genes from the ER dependent differential co-expression network in breast cancer cell lines and sorted blood datasets. Figure S9. Immune infiltration in the TCGA breast cancer cohort estimated from the RNAseq data using the signatures from CIBERSORT using the singscore gene set scoring method (top) and from image analysis of H&E stained slides of samples by Saltz et al. for the 7 cell types (bottom). Figure S10. Association between naïve CD4+ T cell infiltration estimates and selected genes from the differential co-expression sub-network containing immune associated genes. [file 13059_2019_1851_MOESM1_ESM.pdf]

Supplementary methods

Score profiles for inference methods

Two bivariate normal distributions are used to model the association between two genes across two conditions. Gene variances are set to 1 and their covariances are σ_1 and σ_2 in each condition respectively. Means are set to 0 for each gene in each condition. n_1 and n_2 observations are sampled from each distribution and concatenated to produce a $2 \times (n_1 + n_2)$ expression matrix. Differential scores are then computed for each method excluding EBcoexpress and the GGM-based method which require more than two genes in the data. Scores are computed for all combinations of correlations in the range -0.9 to 0.9 and range normalised to the interval $[0,1]$ for visualisation purposes. This analysis is performed under two scenarios: balanced proportions where $n_1 = n_2 = 250$ and unbalanced proportions where $n_1 = 350$ and $n_2 = 150$.

Algorithm for generating "true" differential networks for a simulation

Pseudocode for the algorithm used to derive the three representations of a true differential co-expression network is shown below.

algorithm truedcnetwork **is**

input: $Inputs$ //set of input genes

$Genes$ //set of all genes

kd //a knockdown gene

G_{reg} //graph representing the regulatory network

output: $TrueDCnets$ //set containing representations of “true” DC networks

(initialise results)

$G_{direct} \leftarrow (\mathrm{vertices}(\mathrm{G_{reg}}), \emptyset)$

$G_{influence} \leftarrow (\mathrm{vertices}(\mathrm{G_{reg}}), \emptyset)$

$G_{association} \leftarrow (\mathrm{vertices}(\mathrm{G_{reg}}), \emptyset)$

(identify differentially regulated targets and edges in the *association* DC network)

$D_t \leftarrow \emptyset$

for each $g \in G\mathrm{enes} \setminus Inputs$:

  for each $i \in I\mathrm{nputs} \setminus \{kd\}$:

    if $\mathrm{Sensitive(kd,g)} = 1$ **and** $\mathrm{Sensitive(i,g)} = 1$:

      $D_t \leftarrow D_t \cup \{g\}$

      $\mathrm{edges}(G_{association}) \leftarrow \mathrm{edges}(G_{association}) \cup \{(i, g)\}$

(identify remaining associations in the *association* DC network)

for each $t \in D_t$:

  $I_{eff} \leftarrow \{i \mid (i, t) \in \mathrm{edges}(G_{association})\}$ //effectors for target t

  for each $g \in G\mathrm{enes} \setminus I\mathrm{nputs}$:

    if $\sum_{x \in I_{eff}} \mathrm{Sensitive}(x, g) = \sum_{x \in Inputs} \mathrm{Sensitive}\,(x, g)$:

      $\mathrm{edges}(G_{association}) \leftarrow \mathrm{edges}(G_{association}) \cup \{(g, t)\}$

(identify the *influence* DC network)

$\mathrm{edges}(\mathrm{G_{influence}}) \leftarrow \{(\mathrm{u},\mathrm{v}) | (\mathrm{u},\mathrm{v}) \in \mathrm{edges}(\mathrm{G_{influence}}) \wedge \mathrm{pathExists}(G_{reg}, u, v)\}$

$\mathrm{edges}(G_{direct}) \leftarrow \mathrm{edges}(\mathrm{G_{association}}) \cap \mathrm{edges}(\mathrm{G_{reg}})$

return $\{G_{direct}, G_{influence}, G_{association}\}$

Supplementary figures

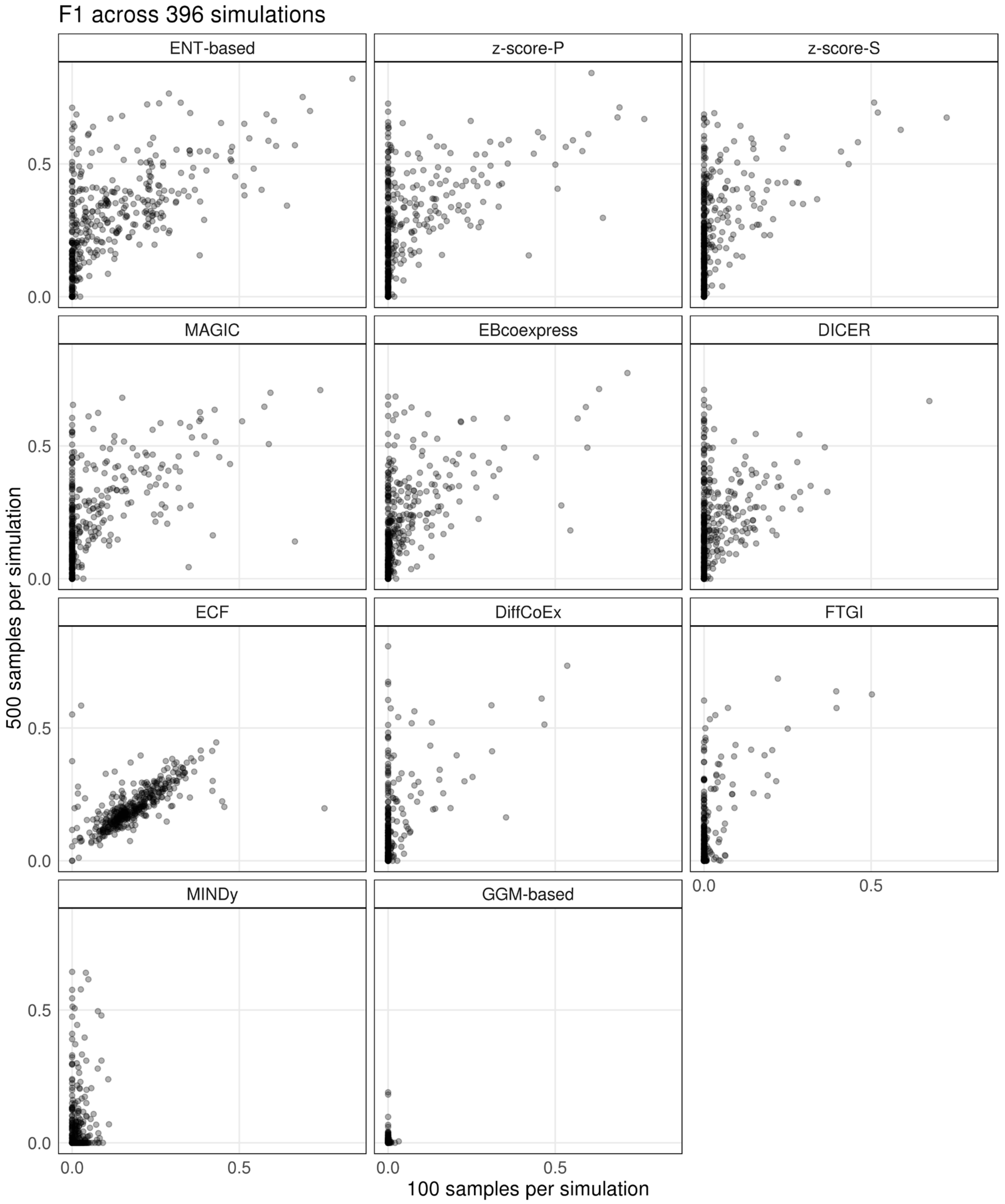

Fig. S1: F1 measure of inference methods across 396 simulations with either 500 or 100 observations. Network and model parameters are the same for both the 100 sample and 500 sample cases but vary across the 396 simulations. Reduction in the number of observations results in a poor performance for most methods excluding ECF.

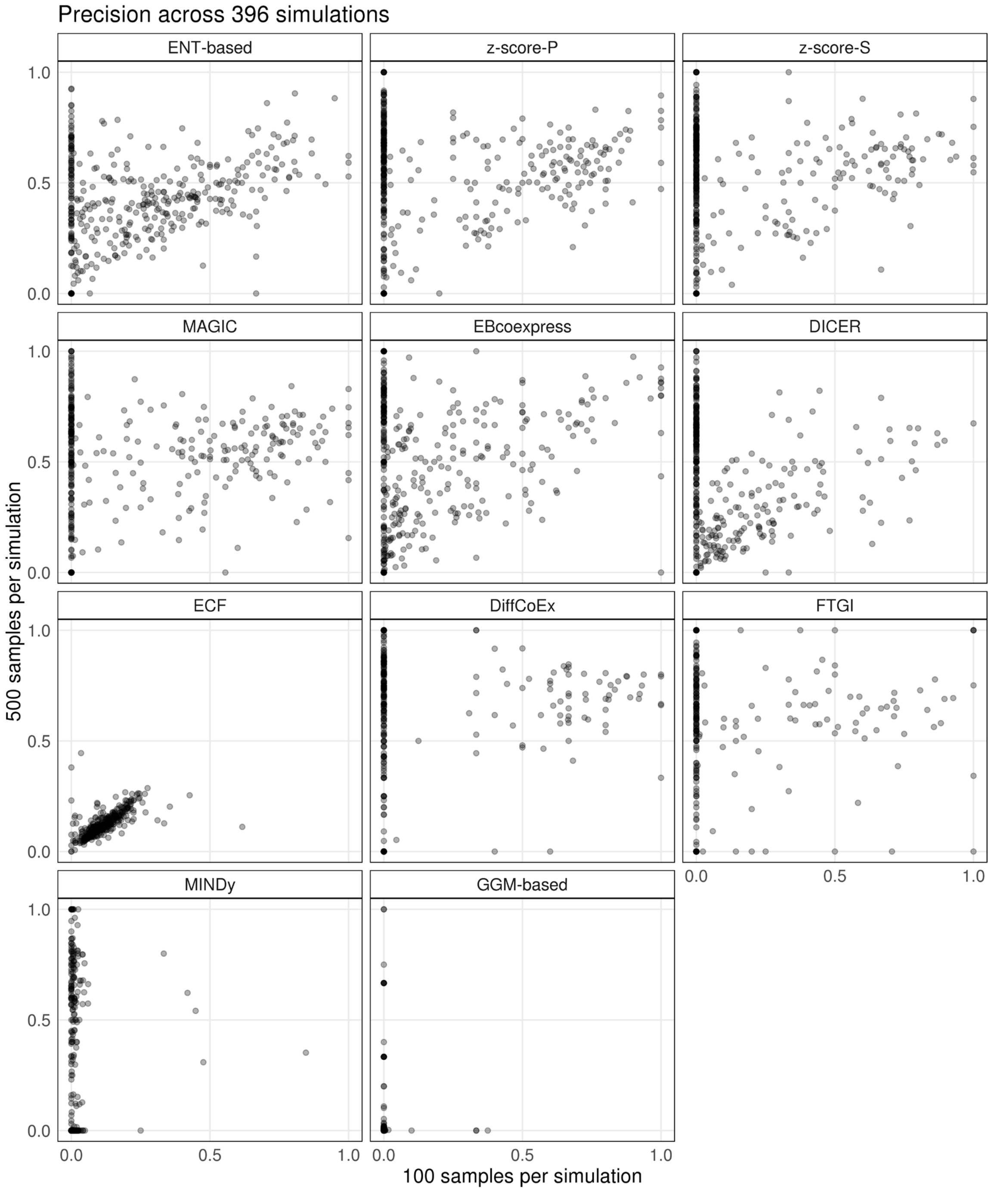

Fig. S2: Precision of inference methods across 396 simulations with either 500 or 100 observations. Network and model parameters are the same for both the 100 sample and 500 sample cases but vary across the 396 simulations. Reduction in the number of observations results in a poorer precision for most methods however the precision of the entropy-based method is retained.

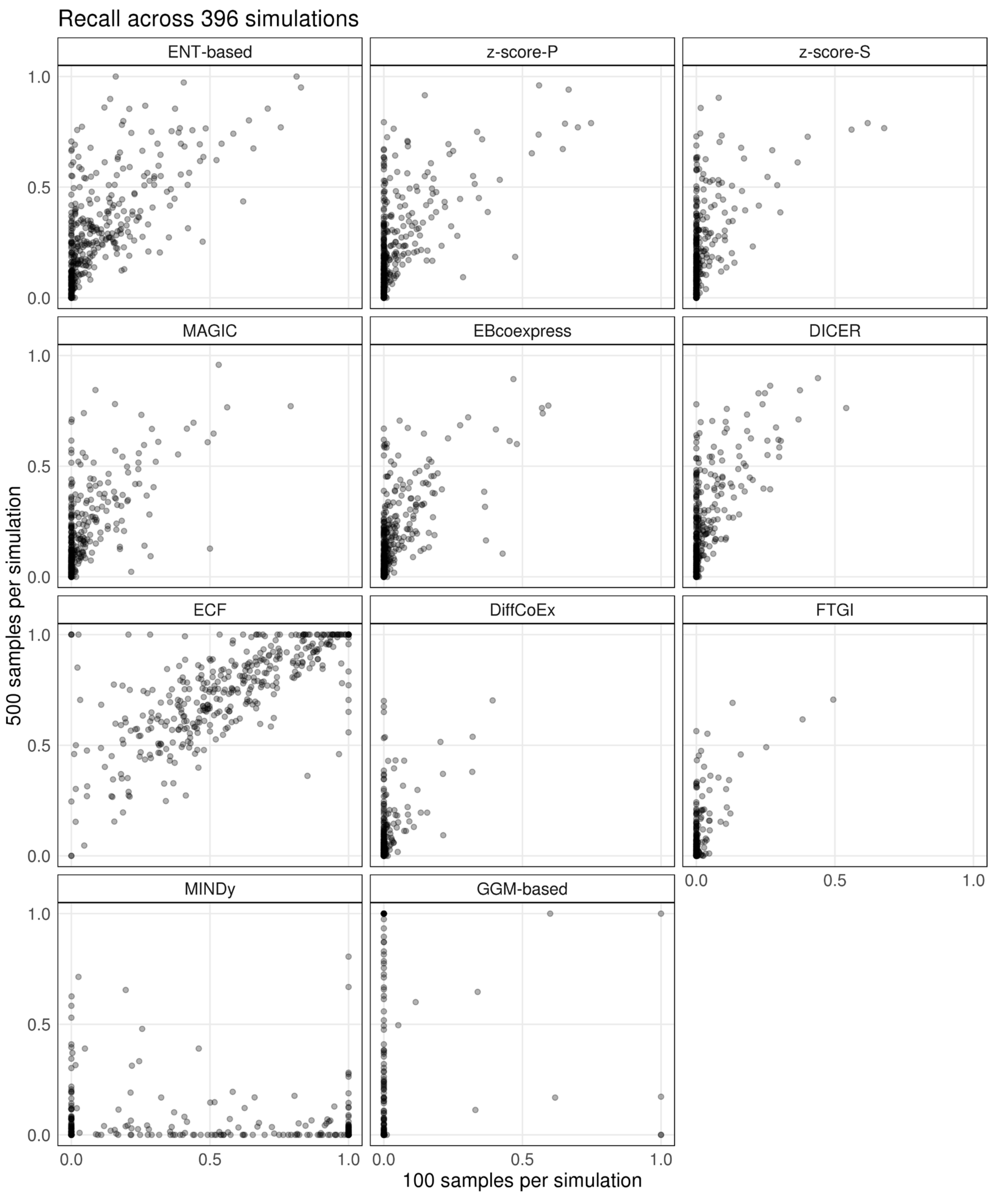

Fig. S3: Recall of inference methods across 396 simulations with either 500 or 100 observations. Network and model parameters are the same for both the 100 sample and 500 sample cases but vary across the 396 simulations. Reduction in the number of observations results in a reduced recall across all methods except for ECF.

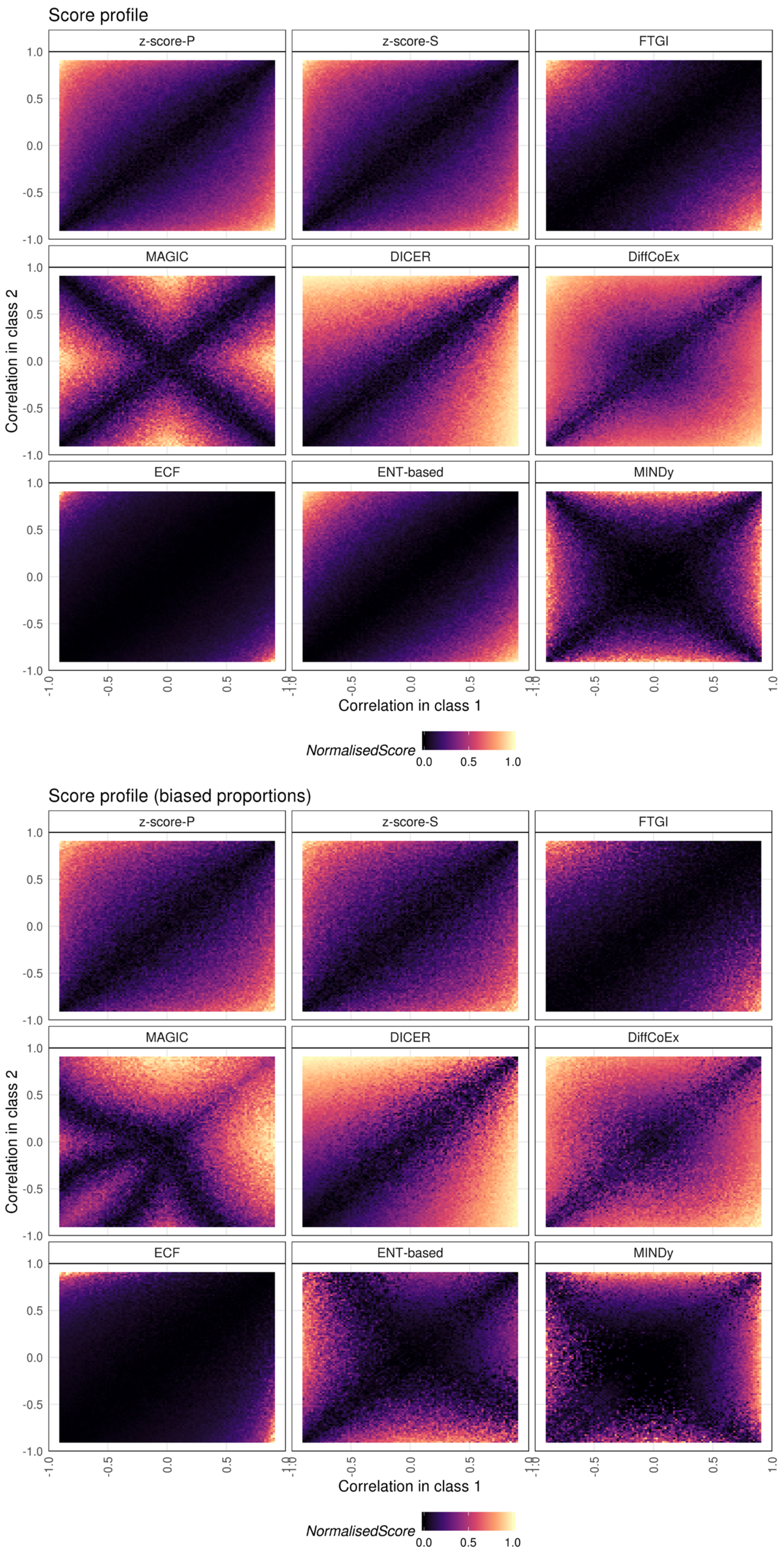

Fig. S4: Score profiles for the different methods without (top) and with (bottom) imbalanced samples in each condition. Scores have been standardised in the interval [0,1] for visualisation purposes. Methods that are invariant to biases in sample biases have a scores symmetric along y = x. MAGIC, ENT-based and ECF are affected by such biases (bottom panel).

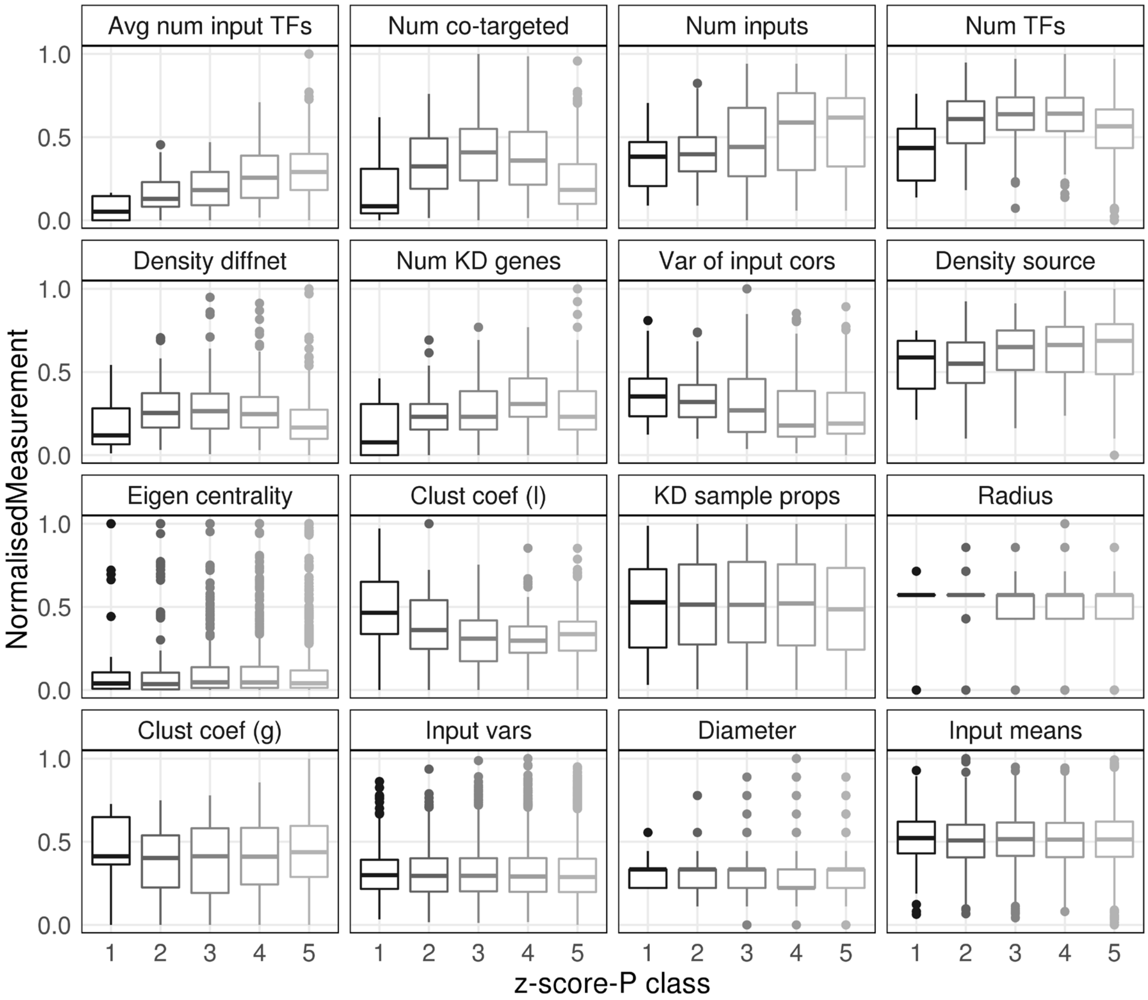

Fig. S5: Classes were determined by hierarchical clustering of the F1 score of the z-score with Pearson's coefficient method across 812 simulations with 1 representing simulations where methods the z-score performed well the best and 5 where performance was poor. Number of samples in classes are 17, 72, 126, 174, 423 from classes 1 to 5 respectively. Normalised measurement represents range transformed (to [0,1]) values per measure. Properties sorted by the F-statistic of an ANOVA test on the classes. See Methods for detailed descriptions of each property measured.

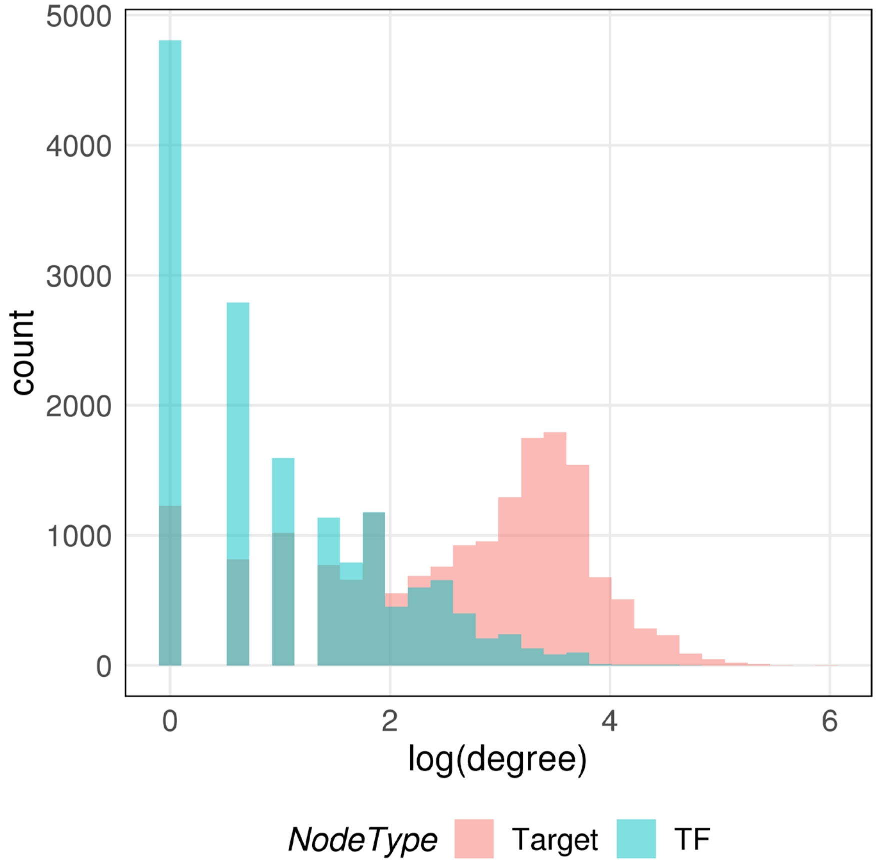

Fig. S6: Degree distribution of target genes and transcription factors. The distribution is calculated across the 812 simulations using the true differential association network. Nodes with no children in the source regulatory network are considered target nodes while all others are considered transcription factors.

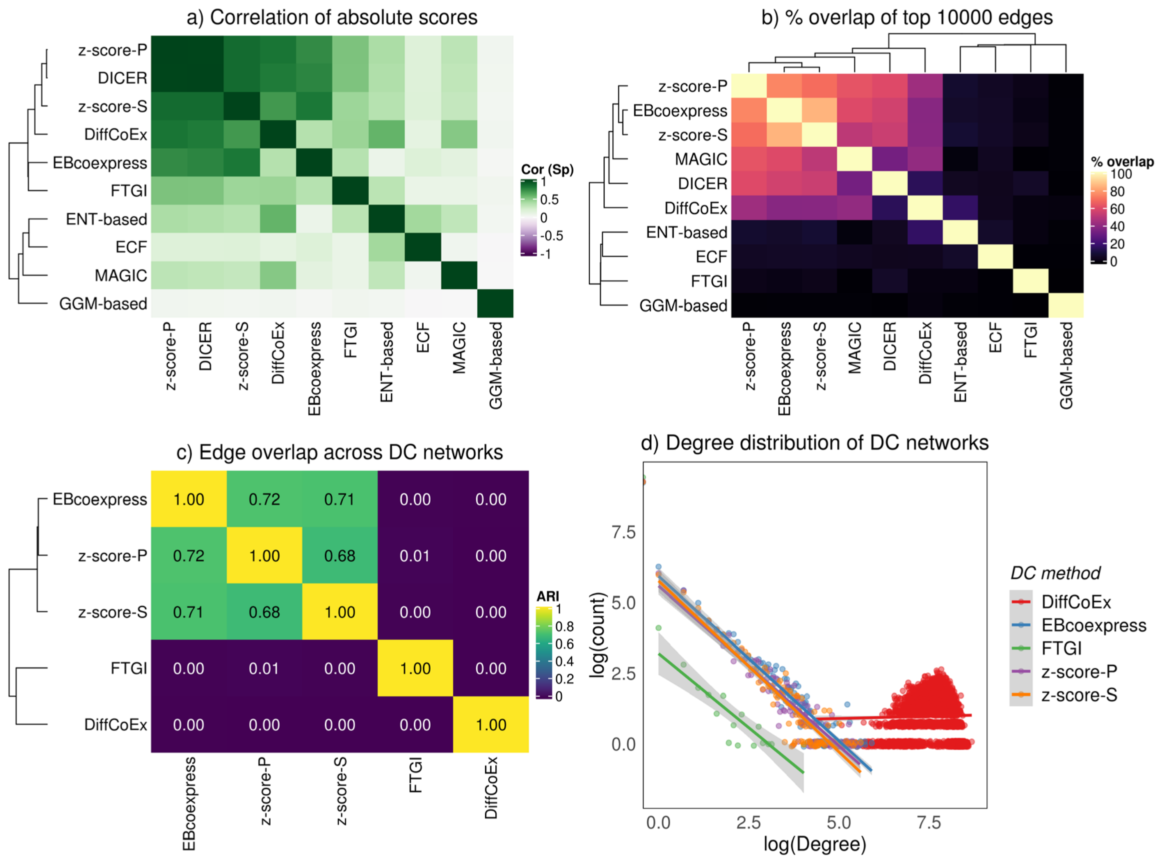

Fig. S7: Comparing differential co-expression scores and networks generated using different inference methods. DC analysis was condition on the ER status and scores/networks generated from the TCGA breast invasive carcinoma RNA-seq data: a) correlation of scores generated by all methods, b) overlap of the top 10000 edges identified from each method, c) adjusted Rand index of networks inferred from methods that completed evaluation within the allocated computational resources, d) degree distribution of edges from these network. A scale-free topology is observed from in the DC networks inferred by methods excluding DiffCoEx, which focuses on identifying modules.

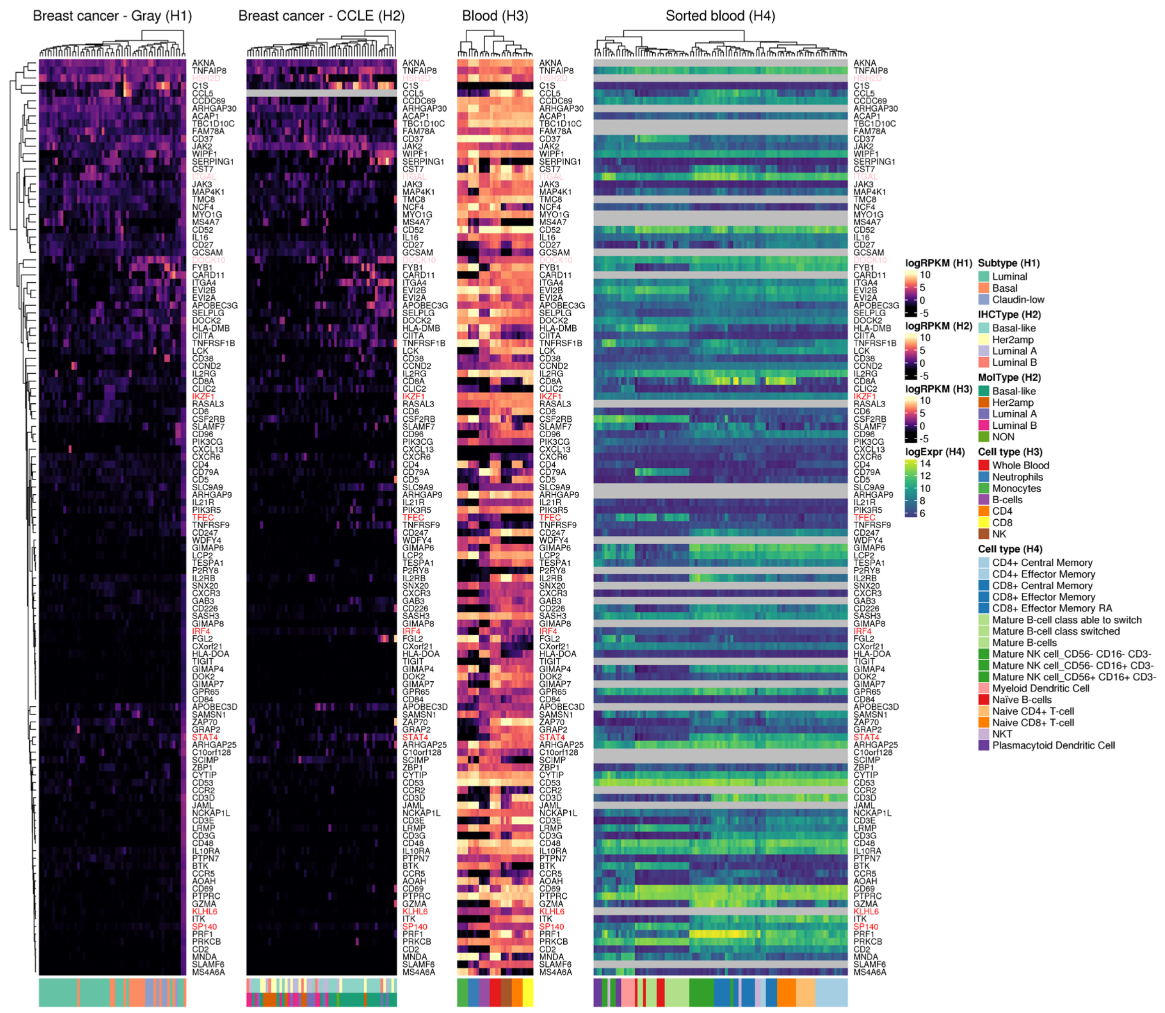

Fig. S8: Expression of genes from the ER dependent differential co-expression network in breast cancer cell lines and sorted blood datasets. From right to left, expression in the Daeman et al. dataset (GSE48213), the cancer cell line encyclopedia (CCLE) dataset, a sorted blood dataset from GSE60424 and a sorted blood microarray dataset from GSE24759. Breast cancer subtypes for the CCLE dataset have been inferred from two independent studies, Neve et al. (IHCType - H2) and Jiang et al. (MolType - H3).

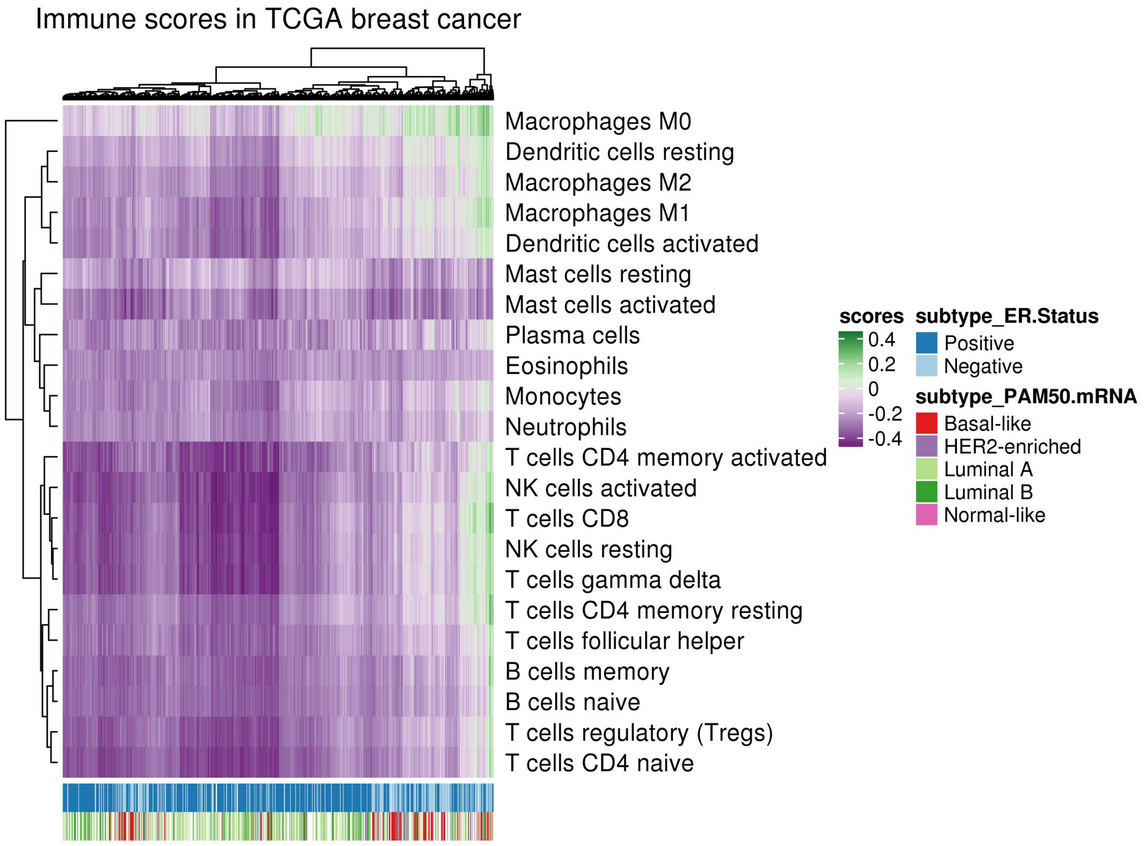

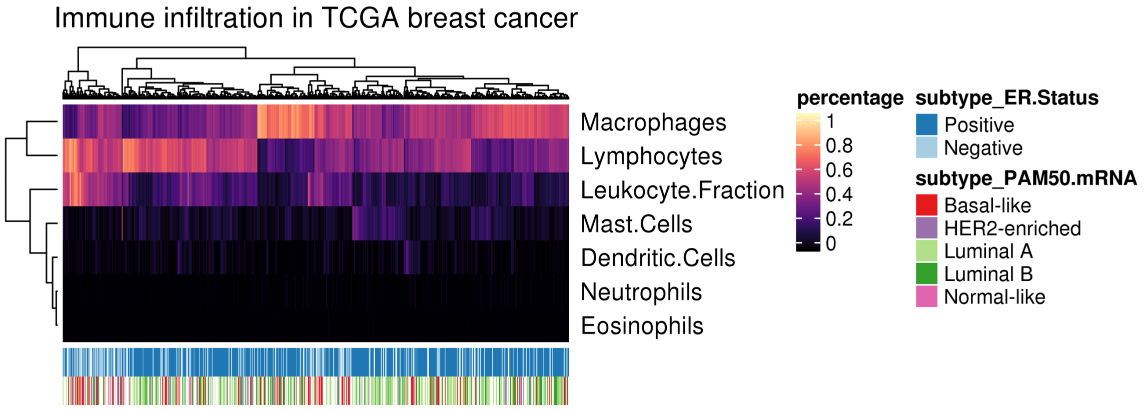

Fig. S9: Immune infiltration in the TCGA breast cancer cohort estimated from the RNAseq data using the signatures from CIBERSORT using the singscore gene set scoring method (top) and from image analysis of H&E stained slides of samples by Saltz et al. for the 7 cell types (bottom).

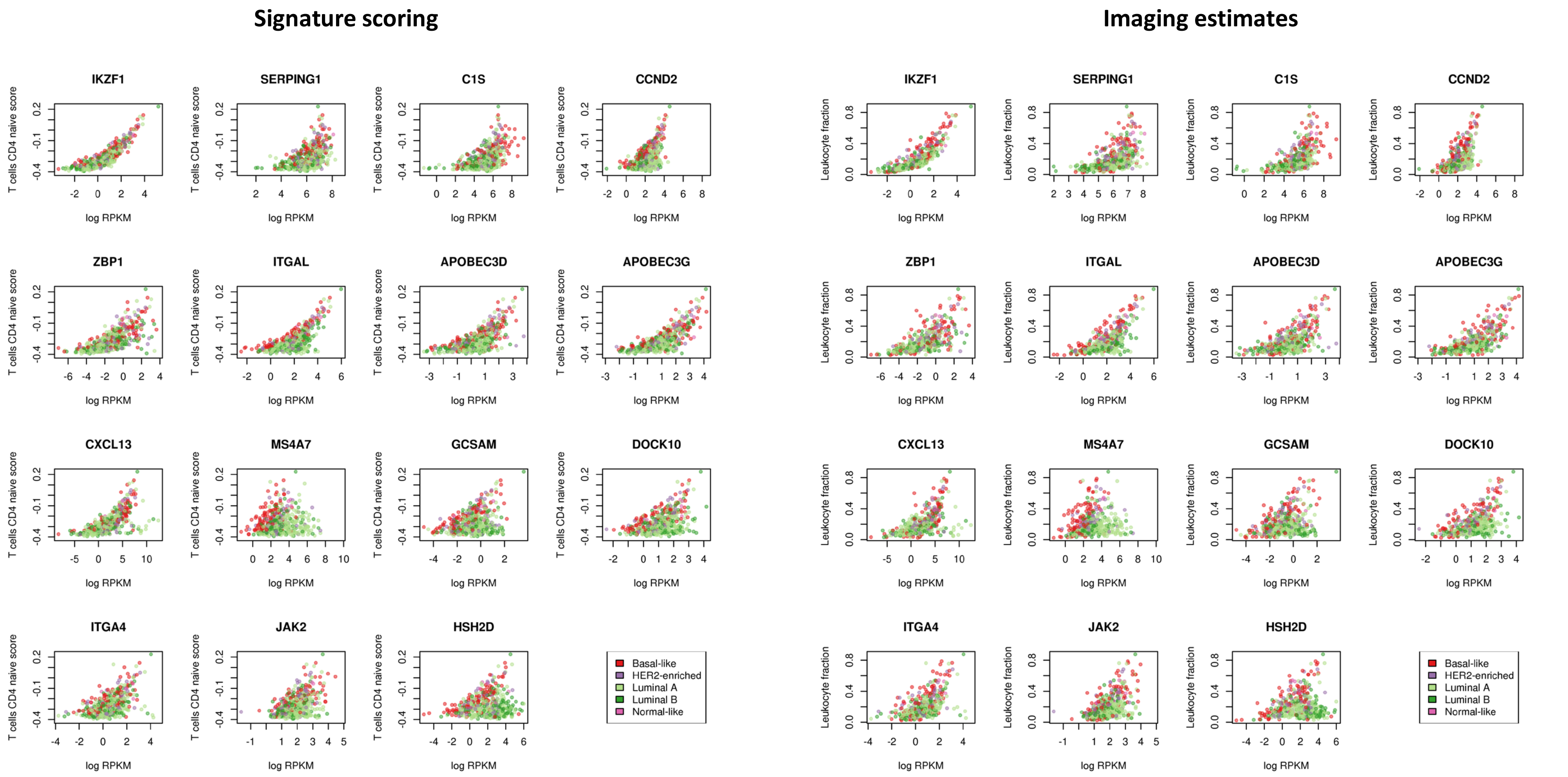

Fig. S10: Association between naïve $CD4^+$ T cell infiltration estimates and selected genes from the differential co-expression sub-network containing immune associated genes. (left) and similar associations with estimates from imaging data (right). Infiltration estimates using the signature scoring approach are shown here. Samples have been annotated with their molecular subtypes and some genes such as HSHS2D show differential association with tumour infiltration (i.e. HSH2D is associated with T cell infiltration in basal-like tumours.
