# Supplementary material for: Differential co-expression-based detection of conditional relationships in transcriptional data: comparative analysis and application to breast cancer
Source: Genome Biol. 2019 Nov 14;20:236. doi: 10.1186/s13059-019-1851-8 (PMC6857226; doi:10.1186/s13059-019-1851-8)
Supplement: Supplementary file 2 — Additional file 2. Review history. [file 13059_2019_1851_MOESM2_ESM.docx]

Review History

**First round of review**

**Reviewer 1**

**Are you able to assess all statistics in the manuscript, including the appropriateness of statistical tests used?**

Yes, and I have assessed the statistics in my report.

**Comments to author:**

In this paper, the Davis and colleagues address the question of reliably detecting differential co-expression. Briefly, they first review multiple

published methods that aim to detect different co-expression and then describe a simulation and evaluation package to compare different

methods to perform differential co-expression detection. They find a top performing method based on simulation and then apply this approach

to a breast cancer dataset. Overall, the paper makes these contribtions: (a) it aims to define differential co-expression based on changes in the regulatory network, which is a more systematic manner than previously done, (b) they provide a software package, dcanr that can be used to try different methods for detecting

differential co-expression, (c) they compare 11 different methods and in the process implement several that are now available within dcanr.

(d) the apply their method to a breast cancer dataset and identify differential networks between ER+ and ER- breast cancer datasets.

Overall, this is a generally clearly written paper, that has several strengths and is providing a utility that could be useful to a large number of investigators.

However the paper is light on methodological novelty or contribution, is limited in scope as it considered only one real dataset

and all the comparisons are largely for pairs of interactions rather than at the module level. Furthermore, there are some aspects of the methods

that need further clarification and description. Specific points follow:

1. The paper reviews different classes of methods for detecting differential co-expression including gene pair based, module based and network based.

However in the experiments for comparing these methods, the specific classes of methods are not incorporated. Ths is important for providing a more

holistic picture of the different methods. The description of how some of the methods were applied (e.g. GGM, DICER and DiffCoex) are not provided. This is important

as some of these methods are module based and may not make inference at the individual pair level.

2. The paper describes three categories of methods, but the differnce between gene-based DC and the network-based is not clear. It is also not clear

what they mean by global influence vs local influence in the description of the gene-based DCs.

3. The paper only evaluates pairwise differential co-expression. It would be even more impactful if we could use this framework to look at

DC in more than 2 conditions.

4. Results in Fig 3 show two types of z-score, but these are not defined. Based on the results it seems that the ENT-based method

is the best. The authors however use the z-score method for application to the breast cancer study. This needs to be better motivated.

It might be even better to show results on real data using the z-score and the ENT-based method.

5. The paper relies on a specific definition of what it means by differential co-expression, namely the change in the network structure

after genetic perturbation of one of the input gene nodes. The authors define the DC association network, DC influence network and the DC direct network.

This is a reasonable model for differeintial co-expression, but it does not consider the case where different conditions represent different

biological samples or tissues in which case a large number of genes could be simultaneously perturbed. Module based analysis could be more informative here.

6. The illustration on Fig 2b which describes the differential interactions could be better described as this is the crux of the definition of

DCs in this paper. Also it might be helpful to provide some pseudocode or additional explanation of how the difference DC networks are obtained.

7. Figure 4 could be moved entirely to supplement, this is not providing a lot of insight. It is also not well explained what the categories of 1-5 (easy to hard) are.

8. The GGM method used is quite rudimentary (but I was missing a better description so I might be wrong here). More recent methods could be considered e.g.

https://www.ncbi.nlm.nih.gov/pmc/articles/PMC5041474/

Minor:

1. Although it is convenient to have one package for generating simulations and algorithms, the simulation is done one time which can be saved.

The different algorithms can be applied easily and independently once the simulated datasets are created.

2. Please clarify the statement "Moreover, regulatory network structures cannot be incorporated therefore propagating effects of differential

regulation cannot be modelled." (line 223-224). GGMs and dependency networks could be constrained to reflect direct regulatory relationships, hence this statement is unclear.

3. Please clarify what global, local and hybrid methods are in the section describing gene based DC methods.

**Reviewer 2**

**Are you able to assess all statistics in the manuscript, including the appropriateness of statistical tests used?**

There are no statistics in the manuscript.

**Comments to author:**

In this paper, Bhuva et al. perform a benchmark of 11 methods to perform differential co-expression analysis (DC) when comparing multiple biological conditions. This approach has been shown to provide increased sensitivity compared to differential expression analysis, and the authors provide example in which the DC would detect signal while the standard DE analysis would not. In particular, DC can provide hibts at regulatory (direct or indirect) links between regulators and target genes. The authors implemented the tested methods within a bioconductor package dcanr, which also provides implementation of several of the described tools.

The topic is of interest, and one can always be grateful when computational groups make the effort to provide a benchmark of existing method with an independent approach. In particular, the question of providing proper benchmarking datasets is crucial, and has been addressed carefully in this work. The authors describe a novel simulation approach, combining sampling of realistic GRN architectures from S. cerevisae with simulation of gene expression using a stochastic approach. Using this simulation allows to distinguish between various levels of regulatory relationships, namely association network, influence network and direct network.

I appreciate the effort of benchmarking and implementation of the methods provided here. The paper is well written and provides ample technical details. I have a number of questions/suggestions:

1. while the overall paper is on method comparison and establishment of a benchmarking strategy, the section on the breast cancer use-case falls somewhat out of this scope. It is not clear how this section relates to the previous ones, as the BC dataset is not used to compare different methods, but to gain biological insights using only one method on the dataset, namely the z-score method. If this paper is on comparing methods, I would suggest to apply the various methods to this dataset and compare the outcomes, rather than focusing on one single method.

2. It would be interesting to complement the study to include methods which have been developed to reconstruct GRN, such as ARACNe or GENIE3 for example. While these methods are not meant to detect DC, they were designed to predict regulatory relationships. As the question of the use of DC to infer regulatory relationships is central in this manuscript, this would provide an interesting additional aspect.

3. I am wondering why the authors chose to use a S. cerevisae GRN, as the topology of yeast GRN and for example human GRN is quite different. Could the authors provide some justification for this or comment on the impact of the topology of the underlying GRN on the simulation results?

**Authors Response**

**Point-by-point responses to the reviewers’ comments:**

We thank the reviewers and the editorial team for their time and feedback. We feel that incorporating this has helped to improve our manuscript. Below we include a point-by-point response to each specific comment.

**Reviewer #1:**

In this paper, the Davis and colleagues address the question of reliably detecting differential co-expression. Briefly, they first review multiple published methods that aim to detect different co-expression and then describe a simulation and evaluation package to compare different methods to perform differential co-expression detection. They find a top performing method based on simulation and then apply this approach to a breast cancer dataset. Overall, the paper makes these contributions: (a) it aims to define differential co-expression based on changes in the regulatory network, which is a more systematic manner than previously done,

(b)   they provide a software package, dcanr that can be used to try different methods for detecting differential co-expression, (c) they compare 11 different methods and in the process implement several that are now available within dcanr.(d) the apply their method to a breast cancer dataset and identify differential networks between ER+ and ER- breast cancer datasets.

Overall, this is a generally clearly written paper, that has several strengths and is providing a utility that could be useful to a large number of investigators.

However, the paper is light on methodological novelty or contribution, is limited in scope as it considered only one real dataset and all the comparisons are largely for pairs of interactions rather than at the module level. Furthermore, there are some aspects of the methods that need further clarification and description.

*We would like to thank the reviewer for their comments. We note that this is a benchmarking study, and as such we feel that the main contribution of the work is in making an evaluation framework for 11 methods (of which 9 had not previously implemented as R packages) available to the research community. The package dcanr can be used to run all 11 methods, alone or in combination. This is a substantial contribution that will advance the adoption of these methods in the field. The real novelty of our evaluation lies in the dissection of the different kinds of “true network” that are present and detectible in expression data, along with a demonstration of how to interpret the biological meaning of inferred networks. To the best of our knowledge, these insights and guidelines are unique to our research, and represent a considerable point of novelty in the conceptualisation of the problem and the interpretation of results from differential co-expression analysis.*

Specific points follow:

1.   The paper reviews different classes of methods for detecting differential co-expression including gene pair based, module based, and network based. However, in the experiments for comparing these methods, the specific classes of methods are not incorporated. This is important for providing a more holistic picture of the different methods.

*All classes of methods are reviewed to provide background for differential co-expression methods. The scope of this study is to benchmark network-based methods only, and by design we do not include module- or gene- based methods. We chose network-based methods because differential co-expression networks can be used to identify differentially co-expressed modules and differentially co-expressed genes, however module- or gene-based methods cannot be used to reconstruct a network. We make this point at line 173-176, and again at line 206-213 (new text below).*

*Networks are much richer in information, and module or gene identification can be performed using different summarisation methods. For instance, differentially co-expressed genes can be identified by computing centrality measures of genes in the differential co-expression network. As such, a highly connected gene in the DC network would have a higher degree/Eigen centrality measure, thus be a differentially co-expressed gene. A similar strategy has been adopted by DGCA [McKenzie et al. (2016) BMC Syst Biol DOI: 10.1186/s12918-016-0349-1] whereby the method MEGENA was used to extract modules from a DC method. Similarly, DiffCoEx and DICER begin by quantifying differential co-expression for all pairs of genes and follow this by extraction of modules.*

*Additionally, the aim of this study is to evaluate method performance in the context of detecting conditional regulatory relationships. Gene- and module-based methods mask information for independent gene pairs and can thus limit interpretation of the underlying regulatory relationships. In contrast, network-based methods retain this information allowing association with true regulatory relationships. These reasons have motivated our choice to limit the scope of our benchmarking to network-based differential co-expression methods.*

*Corresponding changes to the manuscript include:*

***Results/Survey of differential co-expression methods*** *(new text underlined,* *deleted text* *with strikethrough):*

*[Lines 206-213]: Network-based methods are flexible and can be used to identify both differentially co-expressed modules, as well as differentially co-expressed genes. In contrast, module- and gene-based methods cannot be used to reconstruct networks, due to the level at which association information is detected and summarised in the methods’ outputs. Since our goal here is to evaluate the ability of methods to reconstruct conditional regulatory networks, in the following evaluation we focus on network-based methods only. Module and gene-based methods all have valuable applications [19, 25, 26, 36, 40], but are not suited for this specific task.*

The description of how some of the methods were applied (e.g. GGM, DICER and DiffCoex) are not provided. This is important as some of these methods are module based and may not make inference at the individual pair level.

*We have modified the relevant methods sections to improve our description. We clarify that the GGM-based method is used exactly as described in the original publication [Chu et al. (2011) BMC Syst Biol DOI: 10.1186/1752-0509-5-89]. Additionally, we state that any downstream analysis steps (e.g. module extraction by DICER and DiffCoEx) are not performed to try and facilitate a fair evaluation of each method’s capabilities at quantifying pair-wise differential associations. DICER and DiffCoEx were included in the evaluations as they both quantify differential associations for pairs of genes prior to module extraction (lines 174-177 of the manuscript).*

***Lines 173-176 from the manuscript:***

*…Due to the independence of modules extracted using de novo identification, both within and between module differential co-expression can be investigated. DICER and DiffCoEx have these properties and can thus be classified as network-based methods by discarding the module extraction phase. …).*

*Corresponding changes to the manuscript include:*

***Methods/ Inferring DC networks using 11 inference methods differential co-expression networks*** *(new text underlined,* *deleted text with strikethrough):*

*[Lines 891-894]: For the graphical Gaussian model (GGM)-based method, models were fit using the GeneNet (v1.2.13) R package (available from CRAN) with the remaining analysis performed as described by Chu et al. [47] and implemented as part of in our R/Bioconductor package dcanr (v1.0.0)*

*…*

*[Lines 906-910]: We aimed to compare benchmark how well each method quantified independent differential associations. As such, downstream analyses such as: module extraction by DICER and DiffCoEx and “minimum modulator support” by MINDy were not performed. Additionally, the output of all methods, excluding EBcoexpress, is a set of p-values for all possible gene pairs.*

2.  The paper describes three categories of methods, but the difference between gene-based DC and the network-based is not clear. It is also not clear what they mean by global influence vs local influence in the description of the gene based DCs.

*We have modified the description of these methods and now clarify that these methods are gene-based because they summarise the extent of network “rewiring” with respect to a gene of interest.*

*Corresponding changes to the manuscript include:*

***Results/Survey of differential co-expression methods*** *(new text underlined,* *deleted text* *with strikethrough):*

*[Lines 128-147]: Gene-based DC analysis methods identify genes that show changes in associations with other genes across the different conditions. They attempt to quantify the extent to which an individual gene is differentially associated with other genes rather than focusing on the nature, or mechanism, of that differential association. Such gene-level signal patterns could arise from transcription factor (TF) loss of function at the protein level (including post-translational modifications), leading to a loss of regulation across some or all target genes [19]. Notably, if this TF had stable RNA abundances across conditions it would not be identified from a DE analysis even though its targets may be differentially expressed. Gene-based methods would identify this TF as strongly differentially co-expressed, with its the targets being weakly differentially co-expressed. Gene-based DC methods are further stratified into global and local gene-based DC methods. Global gene-based methods quantify differential association of a gene in the context of all other genes, that is, how differentially associated is the gene of interest to every other gene. Local gene-based methods differ in the context of genes whereby differential association is quantified against a subset of genes; for example, genes that are associated to the gene of interest in at least one condition. One sub-class of these methods identify global changes in network topology induced by changes in a gene of interest, including: Global gene-based methods include DCglob [19], the N-statistic [20], differential PageRank centrality [21], and differential Eigen centrality [22]. The other major class of methods identify local changes in structure induced by changes in a gene of interest, including Local gene-based methods include DCloc [20], DCp [24], DCe [24], DiffK [5], differential degree centrality [25], differential motif centrality [22], RIF [26], and metrics based on correlation vectors [27]. DiffRank is a hybrid of these classes where both local and global measures of changes in topology differential association are computed for each gene [28].*

3.    The paper only evaluates pairwise differential co-expression. It would be even more impactful if we could use this framework to look at DC in more than 2 conditions.

*Knockout/knockdown experiments are performed to enable detection of conditional regulatory relationships. These experiments typically result in binary conditions therefore DC inference methods have typically, including here, evaluated with binary conditions.*

*Preference for binary conditions would extend to complex conditions such as tissue type, as the ability to interpret changes is limited when many groups are being compared. DCs across groups could be identified but the specific group where a DC is inferred would not, similar to the problem of applying ANOVA to test for differential expression across many groups. The five methods capable of inferring DC across multiple conditions are ECF, COSINE, DiffCoEx, FTGI and DICER. ECF and by extension COSINE (which uses ECF) compute a statistic summarising differential co-expression across all pairs of conditions. DiffCoEx detects changes in co-expression for each group against the average co-expression across all groups and combines these statistics into a single measure. DICER and FTGI summarise differential co-expression between each condition and a selected reference condition. As such, the statistic changes based on the selected reference condition. In all these cases, DC between a pair of genes is summarised across a set of compared conditions. Consequently, DC may be detected if present but the conditions between which a DC is observed will not be detected, limiting interpretation with respect to the biological system underpinning these changes. Additionally, as the hypotheses across these methods are different, performing a direct comparison is not feasible.*

*Corresponding changes to the manuscript include:*

***Discussion*** *(new text underlined,* *deleted text with strikethrough):*

*[Lines 746-755]: In this benchmarking study we have focused on the analysis of differential co-expression between two conditions. The scenario where DC is detected across multiple conditions is an interesting one, however it presents many complexities. Of the methods examined here, only five (ECF, COSINE, DiffCoEx, FTGI and DICER) allow for multiple conditions. With the exception of ECF and COSINE, they do so by constructing a pairwise comparison, where each group is compared against the average of the others, or a selected reference condition. ECF and COSINE perform a series of pairwise comparisons and aggregate the statistic, in a process analogous to ANOVA. Thus, there is a clear need for the development of new methods that deal with truly multiple comparisons in a way that preserves information about the nature of the differences across conditions.*

4. Results in Fig 3 show two types of z-score, but these are not defined.

*We have added text to clarify that the two types of z-scores are obtained using either Pearson’s or Spearman’s correlation measures and are referred to as z-score-P and z-score-S respectively.*

*Corresponding changes to the manuscript include:*

***Results/Evaluation of inference methods using simulated data*** *(new text underlined,* *deleted text with strikethrough):*

*[Lines 385-388]: We compared 11 differential co-expression inference methods by applying them to 812 simulated datasets (details in Methods). For the z-score method, we computed correlations using Pearson’s and Spearman’s methods therefore two sets of results were generated. These are hereafter referred to as z-score-P and z-score-S respectively.*

Based on the results it seems that the ENT-based method is the best. The authors however use the z-score method for application to the breast cancer study. This needs to be better motivated. It might be even better to show results on real data using the z-score and the ENT-based method.

*We had previously explained (lines 446-455) that ENT-based scores are biased when sample sizes across the conditions are significantly different. We also mention that this is relevant in biological analyses such as ER-status dependent analyses where the proportions of samples are heavily biased (1:3 ratio of ER negative to ER positive samples). This is shown using a simple simulation described in the supplementary methods (section: Score profiles for inference methods) and its results shown in supplementary figure 9. To make our choice of method clear, we have moved this text from the section Results/Dissecting method performance to Results/Evaluation of inference methods using simulated data (changes below).*

*We note our previous recommendation around use of the ENT-based method related to its use as part of an ensemble approach. As we have not performed a complete evaluation of ensemble methods, and to try and prevent reader confusion around this, we have removed corresponding results from this manuscript (removed text is shown below).*

*Corresponding changes to the manuscript include:*

***Results/Evaluation of inference methods using simulated data*** *(new text underlined,* *deleted text with strikethrough):*

*[Lines 446-455]: Though the entropy-based method performs relatively well across most simulations, inferences can be biased by sample size differences. Investigations into the effect of sample size differences revealed that the entropy-based method and MAGIC were influenced by differences in the number of observations across groups (see Supplementary methods and Supplementary figures 4). Biases in the number of samples in each condition are common in biological data, for example, the number of estrogen receptor positive (ER+) samples in clinical breast cancer data are usually three times greater than the ER- samples. In such cases, a method invariant to the differences in proportions is needed. Therefore, despite the slightly better performance of the entropy-based method, these results suggest that the z-score based method is a better and more robust choice for generic applications, particularly when there is a class imbalance.*

*…*

*Due to the extreme properties of the ECF network, it could potentially be used in ensembles to improve prediction by compensating for the errors made by other methods. An improvement would only arise if the errors made by different methods in the ensemble are independent. We therefore sought to investigate the dependence structure of method performance by using the combined results from all 812 simulations. A potential ensemble should include the best performing method therefore the performance of methods across simulations was compared against that of the entropy-based method as a baseline. If a method outperformed the entropy-based method across a subset of simulations, it would signify independence of errors made by that method for the specific subset of simulations. This comparison revealed that the z score with Pearson’s coefficient and ECF outperformed the entropy-based method in 288 and 244 simulations respectively. Improved performance from the ECF method was mainly attributed to a higher recall rate and its effects were specifically across those simulations where the entropy-based method performed poorly. Similar observations were made with the z-score (with Pearson’s coefficient) method as a baseline where ECF and the entropy-based method had better performance across a subset of the simulations (Supplementary figure 4-5), suggesting that these may be viable combinations of methods for an ensemble approach.*

***Results/Dissecting method performance*** *(new text underlined,* *deleted text with* *strikethrough):*

*Figure 4 shows the range of normalised values for each property with classes derived using the F1 score from the z-score method with Pearson’s coefficient. A similar analysis of the entropy-based method is shown in Supplementary Figure 6. Results are consistent for both methods except for the effect of group/class proportions across conditions. The z-score based method is invariant to changes in proportion between the two classes whereas performance was altered for the entropy-based method. This could be explained by the fact that the z-score considers the number of observations in each condition whereas the entropy-based method does not. As such, the entropy-based method may be affected by biases in sample proportion/frequency. Further investigation revealed that the entropy-based method and MAGIC were influenced by differences in the number of observations across groups (see Supplementary methods and Supplementary Figures 8-9). Biases in the number of samples in each condition are common in biological data. For instance, the number of estrogen receptor positive (ER^+^) samples in clinical breast cancer data are usually three times greater than the ER^-^ samples. In such cases, a method invariant to the differences in proportions is needed. Therefore, despite the slightly better performance of the entropy-based method, these results suggest that the z-score based method is a better choice for generic applications and thus we evaluate properties of this approach for the remaining analyses.*

5.   The paper relies on a specific definition of what it means by differential co-expression, namely the change in the network structure after genetic perturbation of one of the input gene nodes. The authors define the DC association network, DC influence network and the DC direct network.

This is a reasonable model for differential co-expression, but it does not consider the case where different conditions represent different biological samples or tissues in which case a large number of genes could be simultaneously perturbed. Module based analysis could be more informative here.

*As discussed in item 1, module-based methods are not in the scope of this study because we specifically want to identify conditional regulatory relationships at a network level.*

*Further, with reference to the point raised by the reviewer regarding the likelihood that biological conditions will have perturbations to more than one of the input genes, most of our simulations knock-down multiple genes (lines 872-874). Some simulations had between 6-10 knockdowns and these were not necessarily independent. This could be thought of as switching entire regulatory programs off, as observed for example, with cellular differentiation towards specific lineages or between cells from different tissue types. The effects of knockdowns propagate downstream in the network and result in 3-57% of input genes being perturbed with consequential effects on a similar proportion of the network. Accordingly, the network prior to any knockdown will differ greatly from that after. As such, we have shown that network-based methods can be applied to detect larger global changes and would result in interpretable results. Interpretations could be extended with module extraction from the differential co-expression network, although, not in the context of understanding conditional regulatory relationships.*

6.   The illustration on Fig 2b which describes the differential interactions could be better described as this is the crux of the definition of DCs in this paper. Also, it might be helpful to provide some pseudocode or additional explanation of how the difference DC networks are obtained.

*Mathematical pseudocode for the algorithm was provided as supplementary material under the section – Supplementary methods/Algorithm for generating “true” differential networks for a simulation. Along with this, we have attempted to provide a better abstraction of the three levels of “true” DC networks we propose.*

*We assume that the reviewer was referring to Figure 2c, which contains the definitions for differential interactions, and have addressed the comment accordingly.*

*Corresponding changes to the manuscript include:*

***Results/Determining differential co-expression for complex networks*** *(new text* *underlined, deleted text with strikethrough):*

*[Lines 330-374]: Larger and more complex regulatory networks show richer patterns of differential co-expression. Figure 2a shows the direct interactions for a randomly sampled network of 150 genes. The network includes 12 input genes, two of which are were selected for perturbation and highlighted in purple and orange in the plot. Expression data was simulated from the network for 500 biological samples, with the two highlighted genes (KD1 and KD2) randomly assigned to wildtype normal or knockdown expression states in each sample (giving four possible combinations for each sample). Figure 2b shows the results of the z-score co-expression DC inference method applied to the expression data. For every gene pair and each knockdown gene, Pearson correlations and Fisher's z-transform were used to test for a correlation difference between the wildtype and knockdown states of each gene knockdown. Correctly predicted differentially co-expressed edges resulting from each gene knockdown were coloured accordingly (purple or orange), and false positives were coloured grey.*

*Next, we analysed the regulatory network to determine which of the empirical correlation differences shown in Figure 2b correspond to regulatory relationships that are genuinely perturbed by the knockdown gene. Such relationships are considered to represent "true" DC and the collection of such relationships is a representation of the "true" DC network. In Figure 2c, we perform a perturbation analysis. In this analysis, we We manipulate the network as a deterministic system without added noise. We perturb each all of the input genes individually (including the two that are selected for perturbation in our simulation experiment – purple and orange) and determine which of the downstream genes are sensitive to the perturbation, that is, show a substantial alteration in response to perturbation of to each a given input gene. This analysis is necessary because not all genes downstream of an input gene are significantly responsive to perturbation of that input gene, especially in cases where the downstream gene has many other upstream input genes. Any input gene that shares a sensitive target with a knockdown gene should manifest DC with that target, as the strength of the association of the input and the target will be different in conditions where the knockdown gene is reduced in expression. We can extend the input gene results to downstream genes that are solely regulated by each input gene, because such genes are tightly correlated with the input gene in the deterministic network. We call this set of DC interactions the association DC network (right left panel of Figure 2c). The association network includes non-causal gene-gene relationships that are "spurious" or "confounded" in the sense that the putative regulator is not upstream of the target gene in the regulatory network but is merely downstream of a causal regulator. Subsetting the association network to gene pairs where the regulato*r *is upstream of the target gene in the network produces the influence DC network (middle panel of Figure 2c). Further subsetting the influence network to gene pairs where the regulator is directly upstream direct interactions (i.e. those in Figure 2a) produces the direct DC network (left right panel of Figure 2c).*

*In essence, these three representations of the “true” network correspond to different levels of information propagation across the network. The direct network represents information flow from a node to an immediate downstream node. The influence network extends this to model information flow from a node to all sensitive downstream nodes for which there exists a path. The association network further includes information shared between nodes due to information flow from a common ancestral node. In this application, we are interested in the changes in information flow resulting from perturbations, and therefore differences in information flow across the regulatory network represent “true” DC networks and we compare all three levels in our evaluation.*

***Supplementary methods/Algorithm for generating “true” differential networks for a simulation***

7.  Figure 4 could be moved entirely to supplement; this is not providing a lot of insight. It is also not well explained what the categories of 1-5 (easy to hard) are.

*We have moved this figure to the supplementary material. Description of the feature being used for clustering has been improved, and characteristics of the extreme classes (1 and 5) are now clearly stated.*

*Corresponding changes to the manuscript include:*

***Results/Dissecting method performance*** *(new text underlined,* *deleted text with* *strikethrough):*

*[Lines 465-474]: Performance was characterised for the z-score with Pearson’s coefficient and entropy-based methods. Simulations were classified based on the F1 score for each method independently obtained from predicting the true DC network. Classification was performed using hierarchical clustering to group the simulations into 5 classes with varying degrees of ‘ability to be inferred’, such that, class label 1 represented those simulations where predictive performance of the z-score was best while class label 5 represented those where performance was poor. Summaries of the different properties were then investigated in these classes., with class label 1 corresponding to simulations where inference methods performed well, and label 5 the simulations where methods performed poorly.*

*The Analysis revealed that the average number of input regulators upstream of each differentially regulated target was the strongest determinant of performance (Supplementary figures 5).*

8.   The GGM method used is quite rudimentary (but I was missing a better description so I might be wrong here). More recent methods could be considered e.g. https://www.ncbi.nlm.nih.gov/pmc/articles/PMC5041474/.

*This is not a method we developed but in fact, one that has been described previously by [Chu et al. (2011) BMC Syst Biol DOI: 10.1186/1752-0509-5-89]. We have simply implemented is as per their description and applied it accordingly. We have clarified this in the text as described in item 1.*

*The method, LDGM, pointed out by the reviewer has now been added to the evaluation [Tian et al. (2016) Nucleic Acids Res DOI: 10.1093/nar/gkw581]. We have added an entry for this method in Table 2. Results of applying this method have been added to Figure 3 of the manuscript. The method requires a regularisation parameter to be tuned for each condition. We perform a binary search within the interval proposed by the original method; searching for a value that results in as many non-zero elements as there are in the “true” differential association network. Details of this have been added to the methods section.*

*We have added an implementation for this method in the dcanr package (version 1.1.4). We have also updated the simulation dataset (version 3 - DOI: 10.26188/5cb8049f593a6) and added precomputed results for this method across all 812 simulations. This will enable future comparisons to use the predictions without re-computing all results. Details of retrieving pre-computed results from using the dcanr package can be found in the documentation at:*

[*https://davislaboratory.github.io/dcanr/articles/dcanr_evaluation_vignette.html#preco mp*](https://davislaboratory.github.io/dcanr/articles/dcanr_evaluation_vignette.html#precomp)

*Corresponding changes to the manuscript include:*

***Results/Survey of differential co-expression methods/Table 2*** *(new text underlined,* *deleted text with strikethrough):*

***Methods/ Inferring DC networks using 11 inference methods differential co-expression networks*** *(new text underlined,* *deleted text with strikethrough):*

*[Lines 894-903]: The minimum and maximum value for the regularisation parameter for LDGM were computed as described by Tian et al. [48]. The parameter was tuned within this interval such that the number of edges in the resulting network matched the average number of edges in the “true” differential association networks resulting from each knockdown. For a knockdown resulting in 100 differential associations, the regularisation parameter would be selected such that the DC network had close to 100 edges. Binary search was performed in the interval to optimise for this parameter for up to 50 iterations. If the parameter was not optimised, the value that minimised the difference between the observed and expected number of edges among the 50 iterations was chosen.*

***Figure 3***

Minor:

1.  Although it is convenient to have one package for generating simulations and algorithms, the simulation is done one time which can be saved.

The different algorithms can be applied easily and independently once the simulated datasets are created.

*This is true and we have done exactly that. The package provides implementations of the evaluation framework (excluding the simulator) and inference methods. Simulated data is provided as a data file that can be read and processed by the package. Results from the evaluations performed in this study are present with these data. As such, the package can be used to perform inference using different methods or simply retrieve pre-computed results (those produced in this work) if a new method needs to be compared against the existing methods. Likewise, any new method can be evaluated against the same simulated dataset.*

*These use cases are demonstrated in section 4 of the package documentation found at* [*https://bioconductor.org/packages/release/bioc/vignettes/dcanr/inst/doc/dcanr_evalua tion_vignette.html.*](https://bioconductor.org/packages/release/bioc/vignettes/dcanr/inst/doc/dcanr_evaluation_vignette.html) *This example uses data from one simulation that is included in the package. Data for all simulations is available from [DOI: 10.26188/5cb8049f593a6].*

2.     Please clarify the statement "Moreover, regulatory network structures cannot be incorporated therefore propagating effects of differential regulation cannot be modelled." (line 223-224). GGMs and dependency networks could be constrained to reflect direct regulatory relationships, hence this statement is unclear.

*This statement refers to strategies previously used to simulate gene expression data with differential co-expression patterns. Previous evaluations used multivariate Gaussian mixture models to simulate expression data where gene-gene dependencies were sampled randomly, without a pre-defined structure of dependence. Graphical models could indeed simulate data from pre-defined networks, however, multivariate Gaussian mixture models will not. We have clarified the statement and specifically stated that we refer to multivariate Gaussian mixture models.*

*Corresponding changes to the manuscript include:*

*Results/Survey of evaluation methods (new text underlined, deleted text with strikethrough):*

*[Lines 247-249]: Moreover, regulatory network structures cannot be incorporated into multivariate Gaussian mixture models therefore propagating effects of differential regulation cannot be modelled.*

3.   Please clarify what global, local and hybrid methods are in the section describing gene-based DC methods.

*Addressed in item 2 of major comments.*

Reviewer #2:

In this paper, Bhuva et al. perform a benchmark of 11 methods to perform differential co-expression analysis (DC) when comparing multiple biological conditions. This approach has been shown to provide increased sensitivity compared to differential expression analysis, and the authors provide example in which the DC would detect signal while the standard DE analysis would not. In particular, DC can provide hints at regulatory (direct or indirect) links between regulators and target genes. The authors implemented the tested methods within a Bioconductor package dcanr, which also provides implementation of several of the described tools.

The topic is of interest, and one can always be grateful when computational groups make the effort to provide a benchmark of existing method with an independent approach. In particular, the question of providing proper benchmarking datasets is crucial, and has been addressed carefully in this work. The authors describe a novel simulation approach, combining sampling of realistic GRN architectures from *S. cerevisiae* with simulation of gene expression using a stochastic approach. Using this simulation allows to distinguish between various levels of regulatory relationships, namely association network, influence network and direct network.

I appreciate the effort of benchmarking and implementation of the methods provided here. The paper is well written and provides ample technical details. I have a number of questions/suggestions:

1.   while the overall paper is on method comparison and establishment of a benchmarking strategy, the section on the breast cancer use-case falls somewhat out of this scope. It is not clear how this section relates to the previous ones, as the BC dataset is not used to compare different methods, but to gain biological insights using only one method on the dataset, namely the z-score method. If this paper is on comparing methods, I would suggest applying the various methods to this dataset and compare the outcomes, rather than focusing on one single method.

*In this section, we wished to demonstrate how we can use insights gained from the evaluation on simulations to interpret results in real biological data.*

*As suggested by the reviewer, looking at the outputs of all other methods would be interesting too, therefore we have performed these additional analyses. We applied all methods to the TCGA breast cancer data to identify differential associations conditioned on the estrogen receptor status. MINDy and LDGM had excessively long compute times and therefore those results could not be presented. We observed strong similarities in the scores generated by methods that use correlation coefficients as a measure of association. This was evident by looking at correlations between the raw scores from each method, overlap between the top 10000 edges from these methods, and by computing the adjusted Rand index between the final networks inferred (supplementary figures 7a-c respectively). Additionally, investigation into the structural properties of the inferred networks showed that networks inferred from FTGI, z-score and EBcoexpress had a scale-free topology (Supplementary figure 7d). We have included a substantial amount of new material covering this new analysis.*

*Corresponding changes to the manuscript include:*

*Results/Application to breast cancer (new text underlined, deleted text with strikethrough):*

*[Lines 535-569]: Differential co-expression analysis of conditioned on the estrogen receptor (ER) status was performed on the TCGA breast cancer data using all DC methods, as described in the Methods using the z-score method revealed 178,487 differential associations between 8,778 genes across ER+ and ER- samples. We filtered out any genes strongly associated with ER (with |correlations| >0.5) to focus on those targets where ER is a co-regulator and not the sole regulator; this is analogous to filtering performed in the simulations. , as performed with the simulations. The analysis could be repeated by setting a more relaxed threshold for discarding ER-regulated genes (we recommend setting this threshold over the interval [0.3, 0.7]). Five methods completed within the allocated computing resources (FTGI, DiffCoEx, z-score-P, z-score-S and EBcoexpress). Scores for 5 more methods were computed but their statistical tests did not complete (DICER, entropy-based, GGM-based, ECF, MAGIC), and 2 methods (LDGM and MINDy) did not generate any results within the allocated time.*

*We first investigated the raw scores to assess similarity between all methods. Absolute scores from methods that use correlation-based measures were themselves highly correlated, with the exception of the entropy-based method (Supplementary figure S7a). ECF and the GGM-based method produced the most distinct scores with very low to almost no association with scores from the other methods. Since statistical tests for some methods did not complete, we used the top 10000 interactions with*

*the highest absolute scores as a proxy for a predicted network. Overlap analysis of these networks reinforced the previous finding of concordance between inferences made using the correlation-based methods (Supplementary figure S7b). The strongest overlap was between networks generated using the z-score with Spearman’s correlation coefficient and EBcoexpress. This observation was further validated by comparing the final predicted networks between these methods, which had both completed within the allocated execution time. An adjusted Rand index (ARI) of greater than 0.7 was observed between the EBcoexpress DC network and the two z-score DC networks (with Pearson or Spearman correlation coefficients). FTGI and DiffCoEx generated distinct networks from these methods as evident by ARIs < 0.02 (Supplementary figure S7c).*

*We then investigated structural properties of the networks from methods that fully completed. Degree distributions of all methods except DiffCoEx followed a power law indicating that these networks had a scale-free topology (supplementary figure S7d), while the DiffCoEx network had many nodes with high degree, however these results may be specific to this dataset. As the inference from both z-score methods and EBcoexpress were highly concordant, we decided to focus further investigations on a representative DC network: the DC network generated from the z-score with Spearman’s coefficient method.*

*The z-score method resulted in a DC network with 178,487 differential associations between 8,778 genes across ER+ and ER- samples. As the resulting network was too large to investigate in full,10−we10 focused our analysis on the most statistically significant*

*interactions (p-value <          	).*

*Methods/TCGA breast invasive carcinoma analysis (new text underlined, deleted text with strikethrough):*

*[Lines 959-970]: All methods were applied to the dataset with the same parameters as those used for simulated data. An adjusted p-values threshold of 1x10^-10^ was applied to generate the DC network. A threshold of 1x10^-10^ was applied on the posterior probabilities generated by EBcoexpress. The regularisation parameter for LDGM was tuned to produce a network with 4700 edges; the average of the number of edges resulting from the two z-score executions (with Pearson’s and Spearman’s coefficient). As some methods were computationally intensive, we allocated 20 processors per method and allowed for a maximum wall time of up to 7 days (up to 3360 CPU hours per method dependent upon the efficiency of parallelisation). The z-score method with Spearman’s correlation coefficient was used to test all pairwise interactions, with a Benjamini-Hochberg correction for multiple testing (with a threshold of 0.0001). Network visualisation was performed using Cytoscape (v3.6), and network analysis used both Cytoscape and the igraph R package (available from CRAN). The RCy3 (v2.0.86) R/Bioconductor package provides a simple, complete interface between R and Cytoscape and was used to load and analyse networks across the two platforms.*

*Supplementary figure 7*

2.   It would be interesting to complement the study to include methods which have been developed to reconstruct GRN, such as ARACNe or GENIE3 for example. While these methods are not meant to detect DC, they were designed to predict regulatory relationships. As the question of the use of DC to infer regulatory relationships is central in this manuscript, this would provide an interesting additional aspect.

*Both co-expression and DC analyses can be used to learn about regulatory networks however the inference made differs greatly. While co-expression analyses aim to infer regulatory relationships, DC analyses aim to identify conditional regulatory relationships. These two forms of analyses, and by extension inferences, are in fact orthogonal. The former focuses on concordant co-expression while the latter discordant/differential co-expression. Though these analyses help uncover regulatory mechanisms, the underlying inferences are not easily comparable. That was why in this benchmarking study, we focused on evaluating DC methods and interpreting results from their application.*

*One way to make these analyses comparable is to use both types to perform the same task. Some gene-based and module-based DC methods compute the co-expression network in each condition independently and compare differences between these two networks. In fact, a naïve approach to determine differential co-expression might be to take the co-expression network or inferred GRN in each condition and look for differences. In this way GRN inference methods could be compared with DC methods in the context of inferring conditional regulatory relationships. We built such DC networks in our simulations using methods that have been shown to perform the best in past GRN inference benchmarking studies [Madhamshettiwar et al. (2012) Genome Med DOI: 10.1186/gm340 & Maetschke et al. (2014) Brief Bioinform DOI: 10.1093/bib/bbt034]. The z-score method by [Prill et al. (2010) PLoS One DOI: 10.1371/journal.pone.0009202] and the most highly cited co-expression analysis method, WGCNA [Fuller et al. (2007) Mamm Genome DOI: 10.1007/s00335-007-9043-3], were used to infer co-expression networks in the independent conditions. Non-overlapping edges between the two conditions formed the inferred DC network. These networks were evaluated against the true DC networks and are shown in the updated figure 3 to perform worse than most DC inference methods. This could be expected as these methods were not designed to infer DC networks. The main problem in using co-expression methods to infer DC networks (which is also discussed in the manuscript: Results/Survey of differential co-expression methods) is that prediction errors are assumed to be independent in each condition thereby resulting in the underestimation of prediction errors. The results additionally discourage the use of co-expression methods to infer of DC networks, despite these methods being the best in their respective application.*

*Finally, as noted by the reviewer, these analyses may be used in conjunction to improve our understanding of the underlying regulatory network and this may indeed pose an interesting question. We too have given this idea much thought, however, concluded it to be an extensive task that would be a natural extension of this study. To achieve this task, we would have to relate both the co-expression and differential co-expression network to the true regulatory network independently. We have been able to map some structures in the DC network back onto the underlying regulatory network though many of these structures need further investigation. Similar analysis needs to be performed for the co-expression network. Following this, we would need to investigate similarities and differences between these networks, as genes with weak differential associations will also be observed in the co-expression network. Such information may aid in improving inference of the underlying GRN, but it requires extensive work, and while we agree it is an interesting extension, we feel that it falls outside the scope of this benchmarking report.*

*Corresponding changes to the manuscript include:*

***Background*** *(new text underlined,* *deleted text with strikethrough):*

*[Lines 69-72]: In contrast to exploring differences across conditions, there are also opportunities to extract functional information from the co-expression of genes [4] (i.e. concordant changes in transcript abundance) [4] using gene regulatory network (GRN) inference methods such as WCGNA [5] and the z-score by Prill et al. [6].*

*…*

*[Lines 87-95]: Both co-expression based GRN analyses and DC analyses can be used to learn about regulatory networks, however the inference made differs greatly. While co-expression analyses aim to infer regulatory relationships, DC analyses aim to identify conditional regulatory relationships. These two forms of analyses, and by extension inferences, are in fact orthogonal. The former focuses on concordant co-expression while the latter discordant/differential co-expression. Though these analyses help uncover regulatory mechanisms, the underlying inferences are not easily comparable. As such, in this benchmarking study, we focused on evaluating DC methods and interpreting results from their application.*

***Results/Evaluation of inference methods using simulated data*** *(new text underlined,* *deleted text with strikethrough):*

*[Lines 385-393]: We compared 11 co-expression inference methods by applying them to 812 simulated datasets (details in Methods). For the z-score method, we computed correlations using Pearson’s and Spearman’s methods therefore two sets of results were generated. These are hereafter referred to as z-score-P and z-score-S respectively. Additionally, we evaluated DC networks generated from co-expression-based GRN methods by taking the difference between co-expression networks identified separately in each condition; WGCNA and a z-score method by Prill et al. [6] were used to generate these co-expression networks. Briefly, approximately 500 expression profiles were simulated from networks with 150 nodes and approximately 2-8 knock downs performed.*

*…*

*[Lines 416-422]: … The GGM-based method, MINDy, and FTGI all performed poorly with the 90^th^ percentile of F1-scores on the association network being lower than 0.25.*

*The 90^th^ percentile of F1-scores on the influence and direct networks were lower than 0.15 for all methods evaluated. As expected, most DC methods outperform co-expression methods (highlighted in Figure 3) at DC inference. Though these methods work well in the task of co-expression analyses, simply taking the difference of co-expression networks does not successfully infer true DC relationships.*

***Discussion*** *(new text underlined,* *deleted text with strikethrough):*

*[Lines 714-724]: We found that the simple z-score method performed the best in benchmarking. Performance of co-expression methods such as WGCNA and the z-score method by Prill et al. [6] was poor in the task of DC inference as could be expected given their development for an alternative application. This demonstrates the need for specialised methods for DC analysis and should discourage the construction of DC networks using the difference between separately inferred co-expression networks, even when the best co-expression analysis methods are used. Additionally, this observation suggests that DC analysis methods, at gene, module, or network level resolution, should refrain from performing inference across the conditions independently and should instead jointly estimate differences between conditions. Validating the choice of the true network through simulation allowed us to identify structures in the differential network that were indicative of the underlying regulatory network structure.*

***Methods/ Inferring DC networks using 11 inference methods differential co-expression networks*** *(new text underlined,* *deleted text with strikethrough):*

*… Precision, recall and the F1 score are were then computed for each method.*

*[Lines 924-932]: Differential co-expression inference was also performed using co-expression based GRN analysis methods. Co-expression networks were generated in the knockdown and wild-type conditions independently and the difference network between the two conditions (i.e. non-overlapping edges) formed the DC network. The WGCNA (v1.68) R package (available from CRAN) was used to run the WGCNA algorithm with default parameters. The co-expression network was generated by selecting all edges with a weight greater than 0.05. The z-score method by Prill et al.*

*[6]  was implemented as originally described. A two-tailed z-test was applied for this method and p-values were adjusted using the Benjamini-Hochberg procedure [73]. An FDR threshold of 0.1 was applied to result in the final co-expression networks.*

***Figure 3*** *(new text underlined,* *deleted text with strikethrough):*

3.  I am wondering why the authors chose to use a *S. cerevisiae* GRN, as the topology of yeast GRN and for example human GRN is quite different. Could the authors provide some justification for this or comment on the impact of the topology of the underlying GRN on the simulation results?

*Since the human gene regulatory network (GRN) is sparsely identified yet, the known GRN can be thought of as being sampled from the full true human GRN. Sampling randomly (i.e. selecting random nodes) will result in differences in topological characteristics of the sampled network from the source network [Marbach et al. (2009) J Comput Biol DOI:*

*GBIO-D-19-00900                                                                                                                                    	Bhuva et al.*

*10.1089/cmb.2008.09TT,  Van den Bulcke et al.  (2006) BMC Bioinformatics  DOI:*

*10.1186/1471-2105-7-43]. Moreover, identification of the human GRN is mostly focused on genes/elements related to diseases which results in biased sampling of the true human GRN. The S. cerevisiae network is more comprehensively realised at this point therefore using it in simulations will result in more biologically accurate networks than using a larger but sparsely realised human network.*

*We have added this as a discussion point in the manuscript.*

*Corresponding changes to the manuscript include:*

***Discussion*** *(new text underlined,* *deleted text with strikethrough):*

*[Lines 671-685]: Studies have discouraged the use of random networks to represent biological networks due to large differences in topological structures [57, 62], thus we sampled networks from the S. cerevisiae regulatory network in a manner which retained biologically relevant motifs and network cliques [56]. Human regulatory networks were not used as they are sparsely identified. Since the human gene regulatory network (GRN) is sparsely identified yet, the known GRN can be thought of as being sampled from the full true human GRN. Sampling randomly (i.e. selecting random nodes) will result in differences in topological characteristics of the sampled network from the source network [57, 62]. Moreover, identification of the human GRN is mostly focused on genes/elements related to diseases which results in biased sampling of the true human GRN. The S. cerevisiae network is more comprehensively realised at this point therefore we assume that using it in simulations will result in more biologically accurate networks than using a larger but sparsely realised human network. The final modelling constraint was the set of logic functions used to model co-regulation. Here, we proposed logic functions derived from co-regulatory mechanisms that are representative of true biological regulation.*

*Other changes:*

*A number of structural changes were made to accommodate modifications made in response to reviewer comments. Excluding modifications in response to reviewer comments (as listed above), a number of small modifications were made to the text. For a full set of changes please refer to the attached document with Track Changes enabled. These include:*

*•         Re-ordering and re-numbering figures in order of appearance in text*

*•         Re-ordering and re-numbering supplementary figures in order of references in text*

*•         Combined some supplementary figures*

*A number of minor changes were made to the text to improve the flow of the manuscript.*

**Second round of review**

**Reviewer 1**

The authors have addressed all my comments.

**Reviewer 2**

The authors have carefully considered the comments and the recommendations for the revision, and included additional analysis (e.g. regarding the application of other methods to the breast cancer dataset). As the results of the different methods applied on this dataset are quite different (with e.g. DiffCoExp having a 0 ARI, fig S7c), I would recommend to add some additional comments on why the authors think that this method in particular behaves strangely (as can be seen also from the degree distribution). That might help readers understand the behavior of some methods on real-world datasets.

**Authors Response**

**Point-by-point responses to the reviewers’ comments:**

Reviewer #2:

The authors have carefully considered the comments and the recommendations for the revision, and included additional analysis (e.g. regarding the application of other methods to the breast cancer dataset). As the results of the different methods applied on this dataset are quite different (with e.g. DiffCoExp having a 0 ARI, fig S7c), I would recommend to add some additional comments on why the authors think that this method in particular behaves strangely (as can be seen also from the degree distribution). That might help readers understand the behavior of some methods on real-world datasets.

*We have added the following two sections of additional text to the Results section to explain why DiffCoEx and FTGI generate different DC networks to z-score and EBcoexpress.*

*Results/applications to breast cancer .*

*We observed an adjusted Rand index (ARI) of greater than 0.7 for comparisons between DC networks generated from the correlation-based methods (EBcoexpress and z-score using either Pearson’s or Spearman’s correlation coefficients). FTGI and DiffCoEx generated distinct networks as evident from ARIs < 0.02 (Additional file 1: Fig. S7c), likely due to differences in how each method calculates association (linear models and soft-thresholded correlation, respectively). ... ... ...*

*While these results may be dataset-specific, we suspect it is because DiffCoEx is originally a module-based inference method. Networks generated using DiffCoEx are intended to be fed into the module detection phase, and therefore tend to be densely connected and possess properties to facilitate module extraction. ...*
